# Supplementary material for: Seroprevalence of SARS-CoV-2 antibodies among Forcibly Displaced Myanmar Nationals in Cox’s Bazar, Bangladesh 2020: a population-based cross-sectional study
Source: BMJ Open. 2022 Nov 21;12(11):e066653. doi: 10.1136/bmjopen-2022-066653 (PMC9679871; doi:10.1136/bmjopen-2022-066653)
Supplement: Supplementary data [file bmjopen-2022-066653supp001.pdf]

Supplemental Material

Seroprevalence of SARS-CoV-2 antibodies among Forcibly Displaced Myanmar Nationals in Cox's Bazar, Bangladesh 2020: a population-based cross-sectional study

Mahbubur Rahman[1*], Samsad Rabbani Khan [1], A S M Alamgir [1], David S. Kennedy [2], Ferdous Hakim [3], Egmond Samir Evers[2], Nawroz Afreen [1], Ahmed Nawsher Alam [1], Md Sahidul Islam [3], Debashish Paul [2], Rijwan Bhuiyan [4], Raisul Islam [2], Adneen Moureen [5], M Salimuzzaman [1], Mallick Masum Billah [1], Ahmed Raihan Sharif [1], Mst. Khaleda Akter [3], Sharmin Sultana [1], Manjur Hossain Khan [1], Kai Von Harbou [2], Mohammad Mostafa Zaman [3], Tahmina Shirin [1], Meerjady Sabrina Flora [6]

[1]Institute of Epidemiology, Disease Control and Research (IEDCR), Dhaka, Bangladesh

[2]WHO Emergency Sub-Office, World Health Organization, Cox's Bazar, Bangladesh

[3]Research and Publication, World Health Organization, Dhaka, Bangladesh

[4]Coordination Center, Ministry of Health and Family Welfare, Cox's Bazar, Bangladesh

[5]IEDCR Field Laboratory, World Health Organization, Cox's Bazar, Bangladesh

[6]Directorate General of Health Services (DGHS), Dhaka, Bangladesh

Contents

Weighted and test adjusted prevalence 2

Bootstrapping for confidence interval 3

Supplementary Table 1. Responses at household, individual and laboratory testing levels, FDMN seroprevalence survey, Bangladesh December 2020 4

Supplementary Table 2: test adjusted over weighted SARS-CoV-2 antibody prevalence by sex across covariates, FDMN population, Bangladesh 2020 5

Supplementary Figure 1. FDMN camps in Ukhiya and Teknaf of Cox's Bazar district, Bangladesh December 2020 6

Supplementary Figure 2: Test adjusted over weighted seroprevalence among respondent occupation, FDMN population, Bangladesh December 2020 7

Weighted and test adjusted prevalence

Primarily we used WANTAI total antibody SARS-CoV-2 ELISA kits to estimate the seroprevalence, which has some limitations and could result in false positives and false negatives estimates. In order to validate the test result, we used Kantaro IgG ELISA test kit. We took the following approach to adjust the seroprevalence of WANTAI by KANTARO kits.

Let $\pi = P(K^{+})$ represent the population prevalence of antibodies to SARS-CoV-2 and let $p = P(W^{+})$ be the proportion of participants who test positive in WANTAI. Let, $s_e = P(W^{+}|K^{+})$ be the sensitivity of the test (the probability of testing positive in WANTAI given an individual is positive in KANTARO), and let, $s_p = P(K^{-})$ be the specificity of the test (the probability of testing negative in WANTAI given an individual is negative in KANTARO). We can write the expected fraction of positive tests, *p*, as follows:

$$p = \pi s_e + (1-\pi)(1-s_p)$$

Therefore, the test adjusted estimated seroprevalence is expressed using following formula [1]-

$$\pi = \frac{p-(1-s_p)}{s_e\,(1-s_p)} \qquad \text{(1)}$$

Of 3,446 total samples in our study, we found 2,090 positives for WANTAI. Again, we used KANTARO quantitative test to those WANTAI positive samples and we found 290 negatives. Since we didn't test any negative WANTAI samples, and we assumed that all negative samples in WANTAI are also negative in KANTARO. A confusion matrix is displayed below-

	Kantaro			
**Wantai**		p	n	Total
	p	1800	290	2090
	n	0	1356	1356
	Total	1800	1646	3046

Table: Confusion Matrix between WANTAI and KANTARO where *'p'* denoting the positive and *'n'* denoting the negative test result.

Sensitivity, $s_e = \frac{1800}{1800+0} = 1.00$, and specificity, $s_p = \frac{1356}{290+1356} = 0.8238$

Therefore, overall seroprevalence, $\pi = \frac{0.574-(1-0.8238)}{1-(1-0.8238)} = 0.4829$

Bootstrapping for confidence interval

We calculated confidence intervals on our weighted sample prevalence estimates based on a non-parametric highest density interval (HDI) bootstrap [2]. Our bootstrap procedure resamples data from our actual datasets for sensitivity, specificity, and prevalence. We use the following steps-

- First, we draw a single bootstrap sample (i.e. with replacement) from the beta distribution of the sensitivity data. For this sample, we calculate the mean value of sensitivity. Let s_{e_j} represent the value for the *jth* iteration of this procedure.
- First, we draw a single bootstrap sample (i.e. with replacement) from the beta distribution of the specificity data. For this sample, we calculate the mean value of specificity. Let s_{p_j} represent the value for the *jth* iteration of this procedure.
- For our weighted estimate, we calculate weighted prevalence of WANTAI in the bootstrap sample . Let p_j represent this value for the jth iteration of this procedure.
- We then calculate the test adjusted prevalence of antibodies to SARS-CoV-2 for the *jth* bootstrap sample using the equation (1)
- Repeat the steps above for j=1...1,000,000 bootstrap samples.
- In our tables, we report the highest density interval (HDI) of the distributions of π_j over the 1,000,000 bootstrap samples as the lower and upper ends of the 95% bootstrap confidence intervals.

The confidence intervals for the test-adjusted estimates were derived using bootstrap sampling, with 1,000,000 parametric bootstrap samples for each estimate, using the "*adjPrevSensSpecCI*" function of the "*bootComb*" R package.

References

1. Chimeddorj B, Mandakh U, Le LV, Bayartsogt B, Deleg Z, Enebish O, Altanbayar O, Magvan B, Gantumur A, Byambaa O, Enebish G. SARS-CoV-2 seroprevalence in Mongolia: Results from a national population survey. The Lancet Regional Health-Western Pacific. 2021 Dec 1;17:100317.
2. Bendavid E, Mulaney B, Sood N, Shah S, Bromley-Dulfano R, Lai C, Weissberg Z, Saavedra-Walker R, Tedrow J, Bogan A, Kupiec T. Covid-19 antibody seroprevalence in santa clara county, california. International journal of epidemiology. 2021 Apr;50(2):410-9.
3. Henrion MY. bootComb—an R package to derive confidence intervals for combinations of independent parameter estimates.

Supplemental Table 1. Responses at household, individual and laboratory testing levels, FDMN seroprevalence survey, Bangladesh December 2020

**Responses**	**All**	
	**n**	**%**
At household level		
Roster completed (RC)	4398	70.9
Vacant house/ No HH respondent available (VH)	152	2.5
House not found*	1112	17.9
Refused interview (RefI)	540	8.7
Total	6202	100
Household response rate (HRR)†		86.4
At individual level		
Completed (C)	3678	83.6
No one eligible*	181	4.1
Unavailable (U)	297	6.8
Refused (R)	242	5.5
Total	4398	100
Individual response rate (IRR)‡		87.2
At laboratory testing level		
Laboratory testing completed (C)	3446	93.7
Sample rejected by laboratory (Rej)	232	6.3
Total	3678	100
Laboratory testing response rate (LTRR)§		93.7
Overall response rate¶		70.6

Note: FDMN, Forcibly Displaced Myanmar Nationals
*Not included as sample as they do not qualify[1] as sample for the Survey
†Household response rate (%)=[RCx100]/[RC+VH+RefI]

‡Individual response rate (%)=[Cx100]/[C+II+U+R+RI]

§Laboratory testing response rate (%)=[Cx100]/[C+Rej]

¶Overall response rate (%)=HRR*IRR*LTRR/(100*100)

[1]The American Association for Public Opinion Research. 2016. Standard Definitions: Final Dispositions of Case Codes and Outcome Rates for Surveys. 9th edition. AAPOR. Available from: https://www.aapor.org/AAPOR_Main/media/publications/Standard-Definitions20169theditionfinal.pdf (Accessed on 3 September 2019)

Supplemental Table 2: test adjusted over weighted SARS-CoV-2 antibody prevalence by sex across covariates, FDMN population, Bangladesh 2020

Variable	Weighted prevalence, % (95% CI)			Weighted & kit adjusted prevalence, % (95% CI)		
	Overall	Female	Male	Overall	Female	Male
Age, years						
All	57.4 (55.1-59.7)	57.4 (54.2-60.6)	57.4 (54.2-60.6)	48.3 (45.3-51.4)	48.3 (44.2-52.3)	48.3 (44.2-52.3)
1-17	49.4 (45.6-53.2)	48.6 (43.0-54.3)	50.2 (45.1-55.2)	38.6 (33.8-43.4)	37.6 (30.7-44.7)	39.5 (33.2-45.8)
18-94	65.5 (63.2-67.8)*	65.4 (62.4-68.2)*	65.7 (62.0-69.3)*	58.1 (55.2-61.1)*	58.0 (54.3-61.6)*	58.4 (53.8-62.9)*
18-49	64.9 (62.3-67.4)	65.1 (61.9-68.1)	64.7 (60.4-68.8)	57.4 (54.1-60.6)	57.6 (53.6-61.4)	57.1 (51.9-62.3)
50-94	68.5 (63.0-73.5)*	67.2 (59.5-74.2)*	69.5 (61.7-76.3)*	61.8 (55.1-68.0)*	60.2 (50.9-68.9)*	63.0 (53.7-71.4)*
Education† (n=3,160)						
No formal education	61.7 (58.8-64.5)	61.4 (57.9-64.8)	62.1 (57.0-67.0)	53.5 (49.8-57.1)	53.1 (48.8-57.4)	54.0 (47.7-60.1)
Primary above	61.8 (58.3-65.2)	59.0 (53.0-64.8)	63.8 (59.5-67.9)	53.6 (49.2-57.9)	50.2 (42.9-57.4)	56.1 (50.8-61.1)
Camp location						
Teknaf	54.8 (48.9-60.6)	59.7 (51.0-67.7)	49.4 (41.3-57.6)	45.1 (37.8-52.2)	51.1 (40.6-60.9)	38.6 (28.7-48.7)
Ukhiya	58.0 (55.5-60.4)	56.9 (53.5-60.3)	59.1 (55.5-62.5)*	49.0 (45.8-52.2)	47.7 (43.3-51.9)	50.4 (45.8-54.6)*
Smoking‡ (n=2,392)						
No	67.5 (64.9-70.0)	65.3 (62.2-68.2)	71.8 (67.1-76.1)	60.5 (57.3-63.7)	57.9 (54.0-61.5)	65.8 (60.1-71.2)
Yes	59.1 (53.9-64.2)*	66.5 (55.6-75.9)	57.5 (51.6-63.2)*	50.4 (43.9-56.6)*	59.3 (46.5-71.2)	48.4 (41.2-55.5)*
BCG vaccination						
No	58.8 (52.7-64.7)	58.9 (50.0-67.2)	58.7 (50.2-66.7)	50.0 (42.5-57.3)	50.1 (39.4-60.4)	49.9 (39.5-59.7)
Yes	57.2 (54.7-59.6)	57.2 (53.8-60.6)	57.2 (53.7-60.6)	48.0 (44.8-51.2)	48.0 (43.8-52.4)	48.0 (43.6-52.3)
Any relevant symptoms						
No symptom	55.3 (52.3-58.2)	53.3 (49.1-57.4)	57.3 (53.2-61.4)	45.7 (41.9-49.5)	43.3 (38.1-48.5)	48.2 (43.1-53.3)
Any symptom	61.2 (57.7-64.6)*	64.6 (59.9-69.1)*	57.5 (52.3-62.5)	52.9 (48.5-57.2)*	57.0 (51.2-62.6)*	48.4 (42.0-54.6)
Any comorbidity						
No comorbidity	56.5 (54.0-58.9)	56.1 (52.6-59.5)	56.9 (53.5-60.2)	47.2 (43.9-50.4)	46.7 (42.3-51.0)	47.7 (43.4-51.9)
Any comorbidity	65.2 (59.2-70.7)*	67.3 (59.8-74.0)*	62.4 (52.4-71.4)	57.8 (50.4-64.5)*	60.3 (51.4-68.7)*	54.4 (42.4-65.5)

Note: FDMN, Forcibly Displaced Myanmar Nationals

*P<0.05

†Among those aged 6 years or more

‡Among those aged 18 years or more

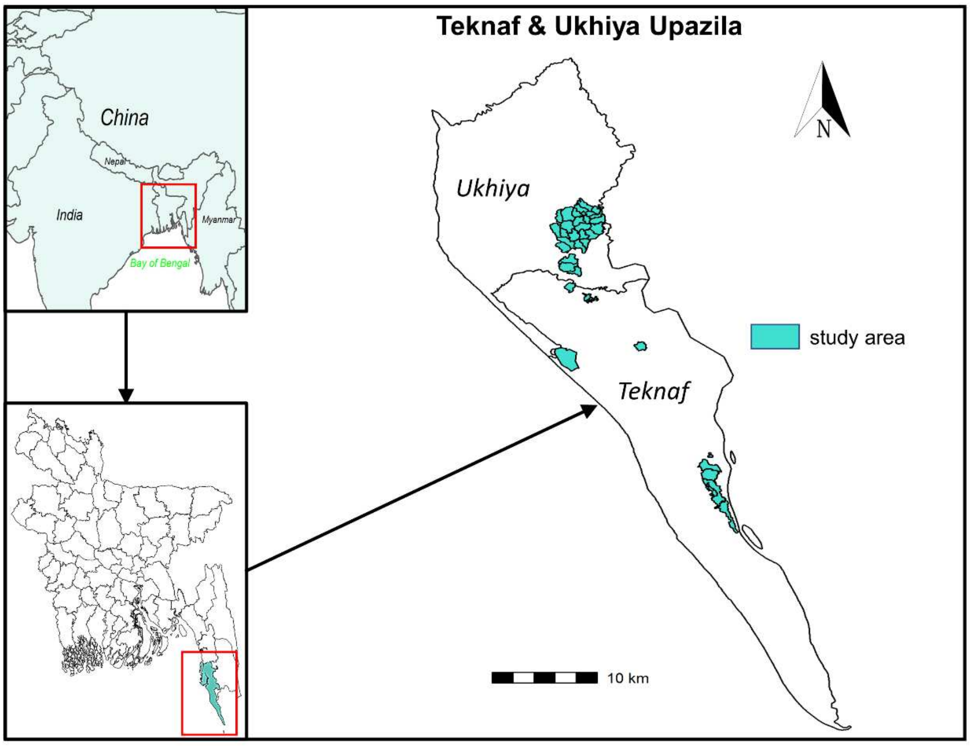

Supplemental Figure 1. FDMN camps in Ukhiya and Teknaf of Cox's Bazar district, Bangladesh December 2020

Note: FDMN, Forcibly Displaced Myanmar Nationals

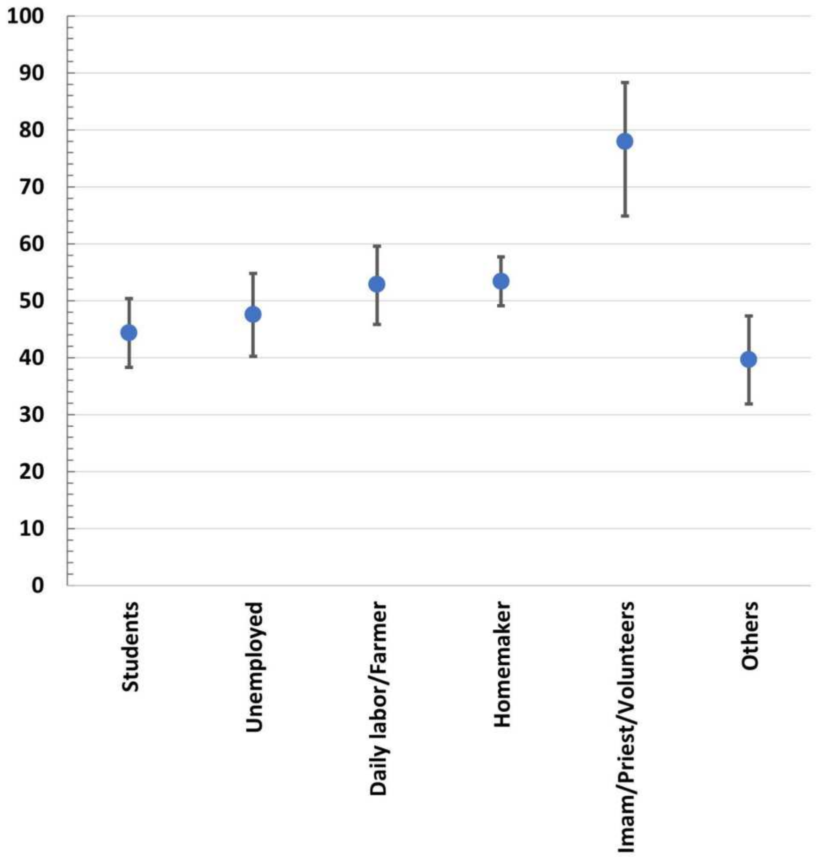

Supplemental Figure 2: Test adjusted over weighted seroprevalence among respondent occupation, FDMN population, Bangladesh December 2020

Note: Blue dot represents the prevalence and error bar indicating confidence interval.
