# Supplementary material for: Seroprevalence of SARS-CoV-2 antibodies among Forcibly Displaced Myanmar Nationals in Cox’s Bazar, Bangladesh 2020: a population-based cross-sectional study
Source: BMJ Open. 2022 Nov 21;12(11):e066653. doi: 10.1136/bmjopen-2022-066653 (PMC9679871; doi:10.1136/bmjopen-2022-066653)
Supplement: Author's manuscript [file bmjopen-2022-066653.draft_revisions.pdf]

BMJ Open

BMJ Open is committed to open peer review. As part of this commitment we make the peer review history of every article we publish publicly available.

When an article is published we post the peer reviewers' comments and the authors' responses online. We also post the versions of the paper that were used during peer review. These are the versions that the peer review comments apply to.

The versions of the paper that follow are the versions that were submitted during the peer review process. They are not the versions of record or the final published versions. They should not be cited or distributed as the published version of this manuscript.

BMJ Open is an open access journal and the full, final, typeset and author-corrected version of record of the manuscript is available on our site with no access controls, subscription charges or pay-per-view fees (http://bmjopen.bmj.com).

If you have any questions on BMJ Open's open peer review process please email info.bmjopen@bmj.com

BMJ Open

Seroprevalence of SARS-CoV-2 antibodies among Forcibly Displaced Myanmar Nationals in Cox's Bazar, Bangladesh 2020: a population-based cross-sectional study

Journal:	*BMJ Open*
Manuscript ID	bmjopen-2022-066653
Article Type:	Original research
Date Submitted by the Author:	15-Jul-2022
Complete List of Authors:	Rahman , Mahbubur ; Institute of Epidemiology Disease Control and Research, Epidemiology<br>Khan, Samsad; Institute of Epidemiology Disease Control and Research, Epidemiology<br>Alamgir, A. S. M; Institute of Epidemiology Disease Control and Research, Virology<br>Kennedy , David ; World Health Organization, WHO Emergency Sub-Office<br>Hakim, Ferdous; World Health Organization Bangladesh, Research and Publication<br>Evers, Egmond; World Health Organization, WHO Emergency Sub-Office<br>Afreen, Nawroz ; Institute of Epidemiology Disease Control and Research, Epidemiology<br>Alam, Ahmed; Institute of Epidemiology Disease Control and Research, Virology<br>Islam, Md. Sahidul; World Health Organization Bangladesh, Research & Publications<br>Paul, Debashish; World Health Organization, WHO Emergency Sub-Office<br>Bhuiyan, Rijwan; Ministry of Health and Family Welfare, Co-ordination Center<br>Islam, Raisul; World Health Organization, WHO Emergency Sub-Office<br>Moureen, Adneen ; World Health Organization, IEDCR Field Laboratory<br>Salimuzzaman , M; Institute of Epidemiology Disease Control and Research<br>Billah, Mallick; Institute of Epidemiology Disease Control and Research, Epidemiology<br>Sharif , Ahmed ; Institute of Epidemiology Disease Control and Research, Epidemiology<br>Akter, Mst. Khaleda; World Health Organization Bangladesh, Research and Publication<br>Sultana , Sharmin; Institute of Epidemiology Disease Control and Research, Virology<br>Khan , Manjur; Institute of Epidemiology Disease Control and Research, Virology<br>von Harbou, Kai; World Health Organization, WHO Emergency Sub-Office<br>Zaman, MM; World Health Organization, Research and Publication<br>Shirin, Tahmina; Institute of Epidemiology Disease Control and Research, Director<br>Flora, Meerjady Sabrina ; Directorate General of Health Services ,

	Additional Director General
Keywords:	COVID-19, Epidemiology < INFECTIOUS DISEASES, Public health < INFECTIOUS DISEASES, EPIDEMIOLOGY, PUBLIC HEALTH

SCHOLARONE™ Manuscripts

I, the Submitting Author has the right to grant and does grant on behalf of all authors of the Work (as defined in the below author licence), an exclusive licence and/or a non-exclusive licence for contributions from authors who are: i) UK Crown employees; ii) where BMJ has agreed a CC-BY licence shall apply, and/or iii) in accordance with the terms applicable for US Federal Government officers or employees acting as part of their official duties; on a worldwide, perpetual, irrevocable, royalty-free basis to BMJ Publishing Group Ltd ("BMJ") its licensees and where the relevant Journal is co-owned by BMJ to the co-owners of the Journal, to publish the Work in this journal and any other BMJ products and to exploit all rights, as set out in our licence.

The Submitting Author accepts and understands that any supply made under these terms is made by BMJ to the Submitting Author unless you are acting as an employee on behalf of your employer or a postgraduate student of an affiliated institution which is paying any applicable article publishing charge ("APC") for Open Access articles. Where the Submitting Author wishes to make the Work available on an Open Access basis (and intends to pay the relevant APC), the terms of reuse of such Open Access shall be governed by a Creative Commons licence – details of these licences and which Creative Commons licence will apply to this Work are set out in our licence referred to above.

Other than as permitted in any relevant BMJ Author's Self Archiving Policies, I confirm this Work has not been accepted for publication elsewhere, is not being considered for publication elsewhere and does not duplicate material already published. I confirm all authors consent to publication of this Work and authorise the granting of this licence.

For peer review only

Seroprevalence of SARS-CoV-2 antibodies among Forcibly Displaced Myanmar Nationals in Cox's Bazar, Bangladesh 2020: a population-based cross-sectional study

Short title: Seroprevalence of SARS-CoV-2 in FDMNs

Authors' list:

Mahbubur Rahman[1*], Samsad Rabbani Khan[1], A S M Alamgir[1], David S. Kennedy[2], Ferdous Hakim[3], Egmond Samir Evers[2], Nawroz Afreen[1], Ahmed Nawsher Alam[1], Md Sahidul Islam[3], Debashish Paul[2], Rijwan Bhuiyan[4], Raisul Islam[2], Adneen Moureen[5], M Salimuzzaman[1], Mallick Masum Billah[1], Ahmed Raihan Sharif[1], Mst. Khaleda Akter[3], Sharmin Sultana[1], Manjur Hossain Khan[1], Kai Von Harbou[2], Mohammad Mostafa Zaman[3], Tahmina Shirin[1], Meerjady Sabrina Flora[6]

Authors' affiliation:

[1]Institute of Epidemiology, Disease Control and Research (IEDCR), Dhaka, Bangladesh

[2]WHO Emergency Sub-Office, World Health Organization, Cox's Bazar, Bangladesh

[3]Research and Publication, World Health Organization, Dhaka, Bangladesh

[4]Coordination Center, Ministry of Health and Family Welfare, Cox's Bazar, Bangladesh

[5]IEDCR Field Laboratory, World Health Organization, Cox's Bazar, Bangladesh

[6]Directorate General of Health Services (DGHS), Dhaka, Bangladesh

*Corresponding author: Mahbubur Rahman

dr_mahbub@yahoo.com

Institute of Epidemiology, Disease Control and Research (IEDCR),

Mohakhali, Dhaka - 1212

Bangladesh

Word Count (excluding title page, abstract, references, figures, tables): 3946

For peer review only

Abstract

Objectives:

The study aimed to determine the seroprevalence, the fraction of asymptomatic infections, and risk factors of SARS-CoV-2 infections among the Forcibly Displaced Myanmar Nationals (FDMNs).

Design:

It was a population-based two-stage cross-sectional study at the level of households.

Setting:

The study was conducted in December 2020 among household members of the FDMN population living in the 34 camps of Ukhia and Teknaf Upazila of Cox's Bazar district in Bangladesh.

Participants:

Among 860,697 FDMNs residing in 187,517 households, 3,446 were recruited for the study. One individual aged one year or older was randomly selected from each targeted household.

Primary and secondary outcome measures:

Blood samples from respondents were tested for total antibodies for SARS-CoV-2 using Wantai ELISA kits, and later positive samples were validated by Kantaro kits.

Results:

More than half (55.3%) of the respondents were females, aged 23 median (interquartile range, 14–35) years and more than half (58.4%) had no formal education. Overall, 2,090 of 3,446 study participants tested positive for SARS-CoV-2 antibody. The weighted and test adjusted seroprevalence (95% confidence interval) was 48.3% (45.3–51.4%) which did not differ by the sexes. Children (aged 1–17 years) had a significantly lower seroprevalence 38.6% (33.8–43.4%) compared to adults (58.1%, 55.2–61.1%). Almost half (45.7%, 41.9–49.5%) of seropositive individuals reported no relevant symptoms since March 2020. Antibody seroprevalence was higher in those with any comorbidity (57.8%, 50.4–64.5%) than those without (47.2%, 43.9–50.4%). Multivariate logistic regression analysis of all subjects identified increasing age and education as risk factors for seropositivity. In children (≤17 years), only age was significantly associated with the infection.

For peer review only

Conclusions:
In December 2020, about half of the FDMNs had antibodies against SARS-CoV-2, including those who reported no history of symptoms. Periodic serosurveys are necessary to recommend appropriate public health measures to limit transmission.

Keywords: COVID-19, Seroprevalence, FDMN, Bangladesh, Pandemic, SARS-CoV-2, Serology, Antibody

For peer review only

Strength and limitations of the study

- This study is one of the largest serosurveys in COVID-19 pandemic among displaced/refugee population.
- Two-stage population-based design of the study with households identified randomly from the Family Counting Number (FCN) using probability proportionate to size of the camps and participants recruited randomly from each selected households with a balanced number of males and females, ensured generalizability and gender equity in data collection.
- Seroprevalence estimates were adjusted to account for test sensitivity and specificity of Wantai test kit validated by Kantaro to address issue of cross-reactivity.
- The household roster could not be completed for all of targeted subjects because the listing of households was done several months before the survey. Non-response at the household level was higher than expected.
- The study outcome relied on testing of antibody response, estimates of which might be affected by the waning antibody levels over time and the ELISA might have cross-reactivity with antibodies to other common human coronaviruses leading to a false-positive result in some instances.

For peer review only

Introduction

A novel pathogen's epidemiological and serological characteristics at the beginning of a pandemic remain uncertain. After the initial detection of severe acute respiratory syndrome coronavirus 2 (SARS-CoV-2) in Wuhan of China in December 2019,[1] surveillance has focused primarily on clinical presentations and severity of COVID-19 disease. A full spectrum of the disease was not evident[2 3] Real-time reverse transcriptase-polymerase chain reaction (rRT-PCR) testing of people can identify patients, including those with mild symptoms. However, previous SARS-CoV-2 infections can be identified using antibody tests, including those who recovered or were not tested being asymptomatic.[4]

The global and regional data on the seroprevalence of antibodies against SARS-CoV-2 ranged during 2020 from 1.4% to 26.0% in countries like the USA,[5 6] Spain,[7] France,[8] Pakistan,[9] India,[10] and Mongolia.[11] The first case of COVID-19 was reported in the Bangladeshi population on 8 March 2020, and the first case in Forcibly Displaced Myanmar Nationals (FDMNs) in Cox's Bazar district on 15 May 2020.[12] One study among Bangladeshi population between April and October 2020[13] estimated the national seroprevalence of immunoglobulins G (IgG) and M (IgM) to be 30.4% and 39.7%, respectively. In Dhaka, the seroprevalence of IgG was 35.4% in non-slum areas and 63.5% in slum areas. In areas outside of Dhaka, the seroprevalence of IgG was 37.5% in urban areas and 28.7% in rural areas. Another study in Bangladesh reported a considerable variation of antibodies in symptomatic and asymptomatic COVID-19 patients.[14] However, seroprevalence estimates in FDMNs are absent, and studies documenting the seroprevalence of SARS-CoV-2 antibodies among displaced populations are limited globally. Bangladesh currently hosts around than one million FDMNs in two sub-districts of a south-eastern frontier district, Cox's Bazar, of Bangladesh.[15]

As of 31 March 2022, 5,902 COVID-19 confirmed cases were reported from the FDMN camps with 42 deaths.[16] These camps are at high risk for COVID-19 due to their very high population density, limited access to improved sanitation , and difficulties in implementing infection control measures.[17] Several outbreaks of communicable diseases have occurred in these camps in the past,[18] including measles, diphtheria and acute watery diarrhoea.

The aim of this survey, which has been adapted from the WHO Unity Study Protocol,[4] was to estimate the seroprevalence of SARS-CoV-2 antibodies and the proportion of

For peer review only

asymptomatic infections among the people living in the FDMN camps in Cox's Bazar district of Bangladesh.

Methods

Setting

We conducted this population-based two-stage cross-sectional survey in December 2020. The study was done on household members of the FDMN population using an adapted version of the WHO Unity Study Protocol.[4] All 34 FDMN camps of Cox's Bazar district were included (**Supplemental Figure 1**). The sampling frame consisted of 860,697 FDMNs residing in 187,517 households registered with the Refugee Relief and Repatriation Commissioner (RRRC)-United Nations High Commissioner for Refugees (UNHCR) database as part of the RRRC-UNHCR Family Counting Exercise (FCE). UNHCR updates its FDMN database periodically collaboratively with the Government of Bangladesh.[19]

The sample size was calculated using an online tool for calculating proportion (OpenEpi).[20] Considering a hypothesized seroprevalence of 2.8%,[5] confidence limits ±0.6%, design effect (1.5) and non-response adjustment (30%), the estimated minimum sample size was 6,202.

In each camp, households were identified randomly by the Family Counting Number (FCN) using probability proportionate to size. Each identified household was assigned either as male or as female to ensure a balanced sample of males and females. One individual aged one year or more was selected from each identified household using a random number generator. Informed written consent was collected from the recruited respondents or their parents or legal guardians in case of children. Written assent was obtained from children aged 12-17 years. People who had any contraindication to venepuncture, such as bruising, swelling, infection, or broken skin, were excluded from the study (**Figure 1**).

Questionnaire and data collection tool

The questionnaire was based on the WHO Unity Protocol[4] and included questions on demographic characteristics and symptom history. The questionnaire was translated into Bangla and the common language used in FDMN camps (Burmese) for participants' better understanding. Pretesting of the questions was carried out and the questionnaire was programmed using KoBo Toolbox[21] running on Android phones and tablets. Phones and tablets were password-protected, and collected data was transferred at the end of each day of the activity.

For peer review only

Field teams and training

The field teams consisted of doctors (team lead), nurses, laboratory technologists, paramedics, and enumerators who were proficient in the local dialect. Forty-nine teams were deployed to collect data. Each field team comprised of two data collectors and one enumerator of the same sex. Female teams collected samples from households assigned as female and male teams collected samples from households assigned as male. In addition, community health workers/ community leaders (M*ajhis)* provided support to the teams to locate the selected households. The field teams' training included interactive discussions on all questions, mock interviews, and blood sample collection exercises.

Campaign to sensitize

Sensitization meetings in all 34 FDMN camps were arranged before initiating data collection by the Camp In-charge assigned by the Government. Support from local religious leaders (e.g., imam), M*ajhis*, site management staff, and community health workers of relevant partner organisations were sought to conduct the study. A flipchart developed by WHO was used to aid these sensitization meetings.

Specimen collection, preservation, and testing procedures

Five milliliters (three ml for young children) of venous blood was collected with aseptic precautions from each of the respondents. The samples were transported to specimen collection hubs maintaining proper labelling and temperature (2-8°C). At the hubs (one for each Upazila), the samples were checked for hemolysis, lipemia and inadequate quantity and sorted and sent to the IEDCR field laboratory at Cox's Bazar Medical College, Cox's Bazar for centrifugation at 3,000 rpm for 20 min and labelled appropriately in aliquots (3 for each individual) for preservation at -80°C.

Samples with laboratory identification numbers were screened for the presence of COVID-19 virus-specific antibodies using Wantai SARS-CoV-2 Antibody ELISA kit[22] according to the manufacturer's instructions. One of the three aliquots was used for ELISA, and the remaining aliquots were stored at -80^{0}C for further testing and quality control.

The blood samples were tested for the qualitative detection of total antibodies (IgG and IgM) to SARS-CoV-2 using a two-step incubation antigen "sandwich" enzyme immunoassay where the receptor-binding domain of SARS-CoV-2 spike protein was present. The patient's serum specimen was added during the first incubation, and a second recombinant SARS-CoV-2 antigen was added during the second incubation, respectively. The microwells were then washed to remove unbound conjugate, and chromogen solutions were added to identify the

For peer review only

amount of antibody captured inside the well. Wells containing specimens negative for SARS-CoV-2 antibodies remained colorless.

The positive test results were validated by Kantaro test kits[23] which is a two-phase ELISA optimized for accurate detection of SARS-CoV-2 IgG antibodies, present in human serum samples with a specificity of 99.8% and a sensitivity of 97.8%.

Quality assurance measures

The data collection procedure and teams were supervised by trained physicians. Independent monitoring visits were made by the investigators and other officials familiar with the study protocol. For external validation and quality control, 5% of the blood samples (positive and negative) were retested using Wantai kits at the IEDCR laboratory in Dhaka, the reference laboratory for SARS-CoV-2 in Bangladesh. Again, positive test results for antibodies to SARS-CoV-2 by Wantai test kits were validated by Kantaro test kits at the IEDCR reference laboratory.

Data weighting and analysis

Data were downloaded from the cloud and merged with laboratory test results with unique identification numbers of respondents. After cleaning of the dataset, weighting for FDMN population[19] was done following the method described by Hakim et al.[24] Base weight was calculated using a probability of selection of respondents among the eligible number of members of the household in a cluster defined by 34 camps, five age groups, and two sexes. Later, base weight was adjusted with non-response weights separately for males and females. The final weight was generated after calibration to frame the population[19] in domains by camp, sex, and age groups. Analysis was done using the final weights.

Categorical variables were reported as number and percentage, and continuous variables by median (interquartile range) or mean (standard deviation) as appropriate. Chi-square was used to test the independence between seropositive and categorical factors.

Our estimation of the seroprevalence of SARS-CoV-2 proceeded in two steps. First, we estimated the weighted seroprevalence of SARS-CoV-2 to represent the FDMN population. Later we used the Rogan-Gladen formula to adjust seroprevalence estimates to account for test sensitivity and specificity of Wantai validated by Kantaro.[25] The 95% confidence intervals for the test-adjusted estimates were derived using bootstrap sampling, with 1,000,000 parametric bootstrap samples for each estimate, using the "adjPrevSensSpecCI"

For peer review only

function of the "bootComb" R package.[26] More details on our bootstrap procedures are available in **Supplemental Material**.

Odds ratios (ORs) and their 95% confidence intervals were obtained using logistic regression analysis to identify the factors associated with weighted test adjusted seroprevalence. Multiple logistic regression models were applied on weighted data for children (1-17 years), adults (18 years and above), and all subjects combined. All variables were entered simultaneously into the multiple regression models. However, smoking could be used for adults only because smoking data were not collected for children. Alpha was set at 5% for considering statistical significance. All analyses were conducted using IBM SPSS version 26.0 and R version 4.1.1.

Patient and Public Involvement

Sensitization meetings conducted in all FDMN camps before initiating data collection to ensure public involvement. Local religious leaders (e.g., imam) and M*ajhis* were involved to implement the study, especially for locating the households.

Patients or the public were not involved in the study design, or reporting, or dissemination plans.

Results

Among the estimated sample size of 6,202, the study teams were successful in locating 4,398 households yielding a response rate of 86.4%. Among the selected households, 3,678 respondents (response rate, 87.2%) completed the individual interview and provided blood samples. Among the collected blood samples, 232 were rejected in the laboratory. Therefore, blood samples from 3,446 respondents completed the laboratory test leading to a response rate of 93.7%. Data from these 3,446 respondents with completed laboratory tests were used for the final analysis. Based on these three response rates, the overall response rate was 70.6%. (**Supplemental Table 1**). All results presented are based on weighted data.

Background information of the study subjects

More than half of the respondents (55.3%) were female. More than two-thirds (69.4%) of them were aged 18 years or above. The median age (interquartile range) of all participants was 23 (14-35) years, which was not different by the sexes. More than half of the school-eligible (age ≥6 years) population reported no formal schooling (58.4%). Most of the respondents reported receiving BCG vaccination (86.3%). Nearly half (42.8%) of adult males (18 years and above) reported smoking tobacco, compared to 7.2% of the females. About one in eight (12.5%) reported at least one comorbid condition. More than one-third (37.0%)

For peer review only - http://bmjopen.bmj.com/site/about/guidelines.xhtml

of the respondents reported having at least one relevant symptom of COVID-19 disease since March 2020. The symptoms included fever, cough, dyspnoea, anosmia, chills, fatigue, sore throat, headache, ageusia, nausea, vomiting, diarrhoea, wheezing, chest pain, rhinorrhoea, conjunctivitis, other respiratory symptoms, muscle aches, myalgia, rash, loss of appetite, nosebleed, and seizures (**Table 1**).

Table 1: Sociodemographic and health related background information of study participants, FDMN population, Bangladesh December 2020 (N=3446)

Variable	Overall	Female	Male
	n (%)	*n (%)*	*n (%)*
Age, years			
All	3446 (100.0)	1906 (100.0)	1540 (100.0)
1-17	1054 (30.6)	472 (24.8)	582 (37.8)
18-94	2392 (69.4)	1434 (75.2)	958 (62.2)
median (IQR)	*23.0 (14.0-35.0)*	*24.0 (18.0-36.0)*	*23.0 (11.0-35.0)*
Education (n=3,160)*			
No formal education	1847 (58.4)	1262 (70.5)	585 (42.7)
Any schooling	1313 (41.6)	528 (29.5)	785 (57.3)
median (IQR)	*0 (0-2)*	*0 (0-1)*	*0 (0-4)*
Camp location			
Teknaf	455 (13.2)	245 (12.9)	210 (13.6)
Ukhiya	2991 (86.8)	1661 (87.1)	1330 (86.4)
Smoking (n=2,392) †	513 (21.4)	103 (7.2)	410 (42.8)
BCG vaccination	2974 (86.3)	1665 (87.4)	1309 (85.0)
Any relevant symptoms‡	1274 (37.0)	723 (37.9)	551 (35.8)
Any comorbidity§	430 (12.5)	268 (14.1)	162 (10.5)

Note: FDMN, Forcibly Displaced Myanmar Nationals

* Among those aged ≥6 years

† Among those age ≥18 years

‡ Symptoms include fever (20.3%), cough (20.2%), dyspnoea (3.1%), anosmia (1.9%), chills (1.6%), fatigue (10.3%), sore throat (3.9%), headache (10.9%), ageusia (4.5%), nausea (3.9%), vomiting (3.9%), diarrhoea (5.8%), wheezing (1.4%), chest pain (2.9%), rhinorrhoea (11.8%), conjunctivitis (1.1%), other respiratory (0.6%), muscle aches (5.4%), myalgia (3.9%), rash (1.8%), loss appetite (3.7%), nosebleed (0.1%), seizures (0.2%)

§ Comorbidities include cancer (0.1%), diabetes (2.5%), hypertension (5.8%), heart diseases (1.3%), asthma (1.9%), chronic lung disease (0.5%), chronic liver diseases (0.9%), thalassemia or other chronic haematological disorder (0.1%), chronic kidney diseases (0.3%), chronic neurological impairment (0.3%), bone marrow diseases (0.5%)

Seroprevalence of SARS-CoV-2

Overall, 2,090 (928 male and 1,162 female) of 3,446 study participants tested positive for SARS-CoV-2 antibody with the Wantai kit (**Table 2**). The overall crude, weighted, and weighted and test adjusted seroprevalence of SARS-CoV-2 antibodies was 60.7% (95% CI: 59.0–62.3%), 57.4% (95% CI: 55.2–59.7%) and 48.3% (95% CI: 45.3–51.4%), respectively. The distribution of infection (weighted and Kantaro test adjusted) was 57.4 (54.2–60.6) and did not differ between males and females. Children and adolescents (age 1–17 years) had lower prevalence (38.6%; 95% CI, 33.8–43.4%) compared to the adults (58.1%; 55.2–61.1).

For peer review only

There was no significant difference in infection between Teknaf and Ukhiya upazila. Non-smoker had significantly higher prevalence (60.5%; 95% CI: 57.3–63.7) compared to smoker (50.4%; 95% CI: 43.9–56.6%). Almost half (45.7%, 95%CI: 41.9-49.5%) of the asymptomatic participants had positive antibody tests. Participants with at least one comorbidity had higher seroprevalence than those with no comorbidity (57.8%; 95% CI: 50.4–64.5% vs 47.2%; 95% CI: 43.9-50.4%) (**Table 2**). This distribution of seroprevalence across covariates were not different between males and females, except camp location where it was significantly higher among males in Ukhiya, lower among smoker males, higher among symptomatic females and higher among females who reported at least one comorbidity (**Supplemental Table 2**).

Table 2. Crude, weighted and test adjusted over weighted prevalence (95% confidence intervals) of SARS-CoV-2 antibody by sex across covariates, FDMN population, Bangladesh December 2020 (N=3446)

Variable	Count	Unweighted	Weighted†	Weight & test adjusted‡
	Positive/Total	% (95% CI)	% (95% CI)	% (95% CI)
Age, years				
All	2090/3446	60.7 (59.0–62.3)	57.4 (55.2–59.7)	48.3 (45.3–51.4)
1-17	547/1054	51.9 (48.8–55.0)	49.4 (45.6–53.2)	38.6 (33.8–43.4)
18-94	1543/2392	64.5 (62.6–66.4)*	65.5 (63.2–67.8)*	58.1 (55.2–61.1)*
Sex				
Female	1162/1906	61.0 (58.7–63.2)	57.4 (54.2–60.6)	48.3 (44.2–52.3)
Male	928/1540	60.3 (57.8–62.7)	57.4 (54.2–60.6)	48.3 (44.2–52.3)
Education§ (n=3,160)				
No formal education	1147/1847	62.1 (59.8–64.3)	61.7 (58.8–64.5)	53.5 (49.8–57.1)
Any education	842/1313	64.1 (61.5–66.7)	61.8 (58.3–65.2)	53.6 (49.2–57.9)
Camp location				
Teknaf	261/455	57.4 (52.7–62.0)	54.8 (48.9–60.6)	45.1 (37.8–52.2)
Ukhiya	1829/2991	61.2 (59.4–62.9)	58.0 (55.5–60.4)	49.0 (45.8–52.2)
Smoking‖ (n=2,392)				
No	1240/1879	66.0 (63.8–68.1)	67.5 (64.9–70.0)	60.5 (57.3–63.7)
Yes	303/513	59.1 (54.7–63.4)*	59.1 (53.9–64.2)*	50.4 (43.9–56.6)*
BCG vaccination				
No	306/472	64.8 (60.3–69.1)	58.8 (52.7–64.7)	50.0 (42.5–57.3)
Yes	1784/2974	60.0 (58.2–61.8)*	57.2 (54.7–59.6)	48.0 (44.8–51.2)
Any relevant symptoms¶				
No symptom	1282/2172	59.0 (56.9–61.1)	55.3 (52.3–58.2)	45.7 (41.9–49.5)
Any symptom	808/1274	63.4 (60.7–66.1)*	61.2 (57.7–64.6)*	52.9 (48.5–57.2)*
Any comorbidity††				
No comorbidity	1800/3016	59.7 (57.9–61.4)	56.5 (54.0–58.9)	47.2 (43.9–50.4)
Any comorbidity	290/430	67.4 (62.8–71.9)*	65.2 (59.2–70.7)*	57.8 (50.4–64.5)*

For peer review only

Note: FDMN, Forcibly Displaced Myanmar Nationals
* P<0.05 for comparisons between seropositives and categorical factors
† Weighted to FDMN Population size (N=860,697) and distribution published by Refugee Relief and Repatriation Commissioner & United Nations High Commissioner for Refuge, 2020.
‡ Rogan-Gladen formula was used to adjust seroprevalence estimates to account for test sensitivity and specificity of Wantai kits validated by Kantaro [25]
§ Among those age ≥ 6 years
‖ Among those age ≥ 18 years
¶ Symptoms include fever, cough, dyspnoea, anosmia, chills, fatigue, sore throat, headache, ageusia, nausea, vomiting, diarrhoea, wheezing, chest pain, rhinorrhoea, conjunctivitis, other respiratory, muscle aches, myalgia, rash, loss of appetite, nosebleed, seizures
†† Comorbidities include cancer, diabetes, hypertension, heart disease, asthma, chronic lung disease, chronic liver disease, thalassemia or other chronic haematological disorder, chronic kidney disease, chronic neurological impairment, bone marrow disease

For peer review only

The prevalence of SARS- CoV-2 antibody was higher in adults than in children, irrespective of their self-report of symptoms (**Figure 2**). However, prevalence was almost the same in two groups of adults (18-49 years and 50-94 years). Among those having comorbid conditions, the highest seroprevalence was found among participants with self-reported chronic liver disease (67.8%) followed by those with bone marrow disease (66.7%), hypertension (60.6%), diabetes (58.9%), heart disease patients (57.1%) and chronic lung disease/ asthma (52.5%) (**Figure 3**). An increasing seroprevalence was observed with the number of self-reported comorbidities, 55.1% with one comorbidity and 71.8% with three or more comorbidities.

Factors associated with anti-SARS-CoV-2 antibody seropositivity

Univariate logistic regression analysis identified increasing age (OR: 1.02; 95% CI: 1.02–1.03), increasing education level (OR: 1.07; 95% CI: 1.03–1.11), history of relevant symptoms (OR: 1.28; 95% CI: 1.06–1.54) and presence of co-morbid conditions (OR: 1.44; 95% CI: 1.10–1.90) as statistically significant factors associated with higher risk of seropositivity, whereas smoking (OR: 0.70; 95% CI: 0.55–0.89) was associated with lower risk of seropositivity. In multiple logistic regression for all subjects adjusted for age, sex, camp locations, BCG vaccination, history of symptoms, and comorbid conditions, the association for higher risk of seropositivity persisted for increasing age (aOR: 1.01; 95% CI: 1.01–1.02) and higher education (aOR: 1.09; 95% CI: 1.04–1.13) only. However, after adding smoking to the model for adults (aged 18–94 years because smoking data were not collected for children), respondents who smoked had a lower risk of infection with SARS-CoV-2 (aOR: 0.63, 95% CI: 0.48–0.83). In addition, respondents who had a history of one or more symptoms had higher odds (aOR: 1.34; 95% CI: 1.08--1.67) of having the infection **(Table 3)**.

Table 3. Risk factors of weighted SARS-CoV-2 infection identified in univariate and multiple weighted logistic regression analysis, FDMN population, Bangladesh December 2020 (N=3446)

Factors	Unadjusted	Adjusted		
		Overall	(Age 1-17 years)	(Age 18-94 years)
	OR (95% CI)	OR (95% CI)	OR (95% CI)	OR (95% CI)
Age (Continuous)	1.02 (1.02–1.03)*	1.01 (1.01–1.02)*	1.09 (1.03–1.15)*	1.01 (0.99–1.01)
Sex				
Female	1	1	1	1
Male	1.00 (0.83–1.20)	1.00 (0.81–1.23)	1.30 (0.89–1.88)	1.05 (0.82–1.34)
Education (continuous)	1.07 (1.03–1.11)*	1.09 (1.04–1.13)*	1.08 (0.97–1.21)	1.06 (1.02–1.11)*
Camp Location				
Teknaf	1	1	1	1
Ukhiya	1.14 (0.88–1.47)	1.10 (0.83–1.46)	1.12 (0.68–1.84)	1.07 (0.78–1.47)
Smoking				
No	1			1
Yes	0.70 (0.55–0.89)*			0.63 (0.48–0.83)*
BCG vaccination				
No	1	1	1	1
Yes	0.94 (0.72–1.22)	0.96 (0.72–1.28)	1.18 (0.55–2.53)	0.85 (0.66–1.12)
Symptoms†				
No	1	1	1	1
Yes	1.28 (1.06–1.54)*	1.20 (0.99–1.47)	1.12 (0.77–1.63)	1.34 (1.08–1.67)*
Comorbidity‡				
No comorbidity	1	1	1	1
One or more	1.44 (1.10–1.90)*	1.10 (0.82–1.48)	1.06 (0.54–2.11)	1.16 (0.85–1.60)

Note: FDMN, Forcibly Displaced Myanmar Nationals
*P<0.05
†Symptoms include fever, cough, dyspnoea, anosmia, chills, fatigue, sore throat, headache, ageusia, nausea, vomiting, diarrhoea, wheezing, chest pain, rhinorrhoea, conjunctivitis, other respiratory, muscle aches, myalgia, rash, loss appetite, nosebleed, seizures
‡Comorbidities include cancer, diabetes, hypertension, heart disease, asthma, chronic lung disease, chronic liver disease, thalassemia or other chronic haematological disorder, chronic kidney disease, chronic neurological impairment, bone marrow disease

For peer review only

Discussions

We report a high seroprevalence of antibodies (total immunoglobulin including both IgG and IgM) to SARS-CoV-2 virus (48.3%) using a cross-sectional survey conducted in December 2020 among FDMNs living in Cox's Bazar district of Bangladesh. This high seroprevalence contrasts with a very low number of infections detected by RT-PCR before the study period. This probably is not surprising as other studies had similar observations even with low seroprevalence, like nationally representative study in Sierra Leone in March 2021 reported weighted seroprevalence of only 2.6%, but it was 43 times higher than the reported number of cases.[27] Seroprevalence study in Pakistan during October to November 2020 although reported a lower seroprevalence of 7.1% based on a low sensitive rapid antigen testing (‘Bioperfectus’ kits for IgG/IgM), the estimated seroprevalence was 62 times higher than the

reported number of cases before the survey period in the sampled districts.[28] Although we found a higher seroprevalence (58.1%) in adults than children aged 1–17 years (38.6%), there was no difference in seroprevalence for older adults (aged 60 and above years) compared with middle-aged adults (aged 40–59 years), which supports the findings of the systematic review by Levin et al.[29] No difference of seroprevalence between sexes was found through our study. Earlier study in Bangladesh reported that females had a higher rate of both IgG and IgM seropositivity compared with males, but the findings were not significantly different.[13] Similar higher seropositivity among females was reported in Sierra Leone, without statistically significant difference.[27] Seroprevalence study in Pakistan also did not report any significant different results among males and females.[28]

Nearly half 45.7% of the people who did not report any symptom since March 2020 also had evidence of SARS-CoV-2 virus infection. They might have had very mild symptoms to recall correctly or were in fact asymptomatic. The younger age profile of the FDMN population also might explain the higher proportion of mild or asymptomatic infection.[30] Because the current study was conducted during a pandemic, the time of the survey, geographic locations, age and sex composition of the sample, and type of antibody assessment should be considered before judging the findings or comparing it with other studies.

The current study examines SARS-CoV-2 infections among those temporarily or chronically experiencing homelessness (or otherwise living in precarious housing) during the COVID-19 pandemic. The FDMN community has intrinsic differences from the host community, especially regarding awareness of COVID-19 issues and access to related information. Although their access to healthcare was beyond the scope of this survey, understanding the risk and needs of this group is crucial to containing the spread of the pandemic.

Our study findings indicated that by December 2020, almost half of the FDMN people were exposed to the SARS-CoV-2 infections, when population vaccination for COVID-19 yet to be started in the country. Another study in Sitakunda subdistrict in the Chattogram division (Cox's Bazar is also located in this division) of Bangladesh conducted in April 2021 has found 63.1% seroprevalence, which was conducted later than our study so expected to have more cumulative seropositive population and sample size, geographical context and type of antibody assessment were different than the current study.[31] The capital city of Dhaka also has a very high population density. Another study[13] reported a seroprevalence of IgG of 35.4% in the non-slum population of Dhaka city between April and October 2020. This study, on the other hand, reported 63.5% seroprevalence in slum areas of Dhaka city. Therefore, the high prevalence we found in FDMN is not unusual in the Bangladesh context. A

For peer review only

seroprevalence study conducted in eight large slums of Mumbai, India in June–July 2020 reported a seroprevalence of 54.1%. However, the same study reported 16.1% seroprevalence in the non-slum areas of Mumbai.[32]

Like others,[6 33] we have observed a lower seroprevalence in children than adults. During the pandemic, children did not go to schools or any other places for an occupation to acquire the infection. Although most adult women stayed at home (because homemaking was their predominant occupation), they had similar infection rates as their male counterparts. Women might have acquired infections at household levels from their husbands or other male members who might have exposure due to contacts with people outside their home. Imam/priests and incentive volunteers had the highest seroprevalence rates in our series (**Supplemental Figure 2**), similar to other studies.[34] Our results also revealed that non-smokers are likely to have higher seroprevalence than smokers. Contrary to the putative effects of smoking on human health, smoking might have suppressed a due immune response to SARS-CoV-2 infections as reported by few other studies,[35-38] although examining this association was beyond the scope for our study. In addition, conflicting associations of BCG vaccination with SARS-CoV-2 infections have been reported. We did not observe any significant association of BCG vaccination. The proposition of a better cellular response in those who have BCG vaccination[39-41] is beyond the scope of our study.

Our study among FDMN population had several limitations. The studies relying on antibody response have their inherent limitations. The estimates reported here might be affected by the waning antibody levels over time. However, it should be noted that vaccination for SARS-CoV-2 had not been initiated in Bangladesh at the time of the study, and therefore was not likely to influence the seroprevalence estimates. The household roster could be completed for about 71% of targeted subjects (**Supplemental Table 1**) because the listing of households was done several months before the survey. Non-response at the household level was higher than expected (13.6%). The population and household level weighting should address the issue of non-response. Again, the ELISA might have cross-reactivity with antibodies to other common human coronaviruses leading to a false-positive result in some instances. Although we were not able to check for cross-reactivity against other coronaviruses, we adjusted seroprevalence estimates to account for test sensitivity and specificity of Wantai validated by Kantaro to address issue of cross-reactivity. Furthermore, self-reports on comorbid conditions and probable COVID-19 symptoms due to a long recall period might impact our study results.

For peer review only

Conclusions

The findings of this study imply that the prevalence of seropositivity is likely to be much higher than the presumed RT-PCR confirmed rates in FDMN population. A high seroprevalence across age and sex groups, irrespective of a history of symptoms, may indicate a inadequacy of infection control measures. Because of a very high population density in the camps, social distancing may not work. Other well-known public health measures, such as face masks and mass vaccination, are warranted.

Data Availability Statement

To protect patients' privacy, the ethical approval obtained for this study prevents human data from being disclosed publicly. Requests to access the datasets should be directed to Professor Tahmina Shirin, Director, IEDCR, email: director@iedcr.gov.bd; directoriedcr@gmail.com.

Ethics statements

Patient consent for publication

Not applicable

Ethics approval

The study protocol was submitted and approved by the Institutional Review Board of the Institute of Epidemiology, Disease Control and Research (Approval Memo No. IEDCR/IRB/2020/24). The participants provided their written informed consent and /or assent (as applicable) to participate in this study. A thumbprint was given if someone was unable to sign.

Acknowledgements

We gratefully acknowledge the field team, MOHFW, DGHS, divisional coordinators, civil surgeons, Upazila health and family planning officers and health assistants for their support. We are also thankful to the following local and international NGO and development partners: WHO, RRRC, UNHCR; Relief International; FH/MTI; ICRC/BDRCS; BRAC; icddr,b; World Bank; Bureau of Population, Refugees, and Migration – United States Department of State through Driving Public Health Action in the World's Largest Refugee Camps; Government of Norway. We are indebted to the camp community leaders (M*ajhis*) to facilitate the data collection and FDMN camp residents for their participation with the study.

For peer review only

Authors Contributions

MR, EE, MMZ, TS and MSF conceptualised this study. MR, ASMA, DSK, FH, EE, ANA, MMZ, TS and MSF developed the methodology and study design. MR, SRK, DSK, FH, EE, NA and MMZ wrote the protocol. MR, SRK, DSK, FH, EE, NA, MMB and MKA developed the data collection tool. DSK and RB developed the software used. ANA, MMZ, TS and MSF validated analyses, data and methodology used. MR, DSK, MSI and RB conducted the formal analysis. MR, SRK, ASMA, DSK, FH, ANA, NA, SS, DP, RB, RI, AM, MS, MMB, ARS, MHK, MMZ and MSF undertook the investigation. ASMA, ANA, KVH, MMZ and TS provided resources for the study. MR, DSK and MSI curated the data. MR, DSK, FH, MSI and MMZ wrote the original draft of the manuscript. MSI designed the visualisations used. MR, SRK, ASMA, FH, ANA, NA, DP, RI, AM, MMB, KVH, MMZ, TS and MSF provided supervision. MR, SRK, ASMA, DSK, FH, NA, DP, RI, AM, MS, MRS, MKA, KVH, MMZ, TS and MSF administered this project. MMZ, TS and MSF acquired the funding for the study. All authors participated in reviewing and editing the manuscript.

Funding

This work was supported by the World Health Organization (Ref. No. 2020/1060317-2). However, no fund has been used to prepare this manuscript.

Competing interests

None declared.

The authors alone are responsible for their views expressed in this article, which do not necessarily represent the institutions' views, decisions, or policies with which they are affiliated.

Patient and Public Involvement

Sensitization meetings conducted in all FDMN camps before initiating data collection to ensure public involvement. Local religious leaders (e.g., imam) and M*ajhis* were involved to implement the study, especially for locating the households.

Patients or the public were not involved in the study design, or reporting, or dissemination plans.

For peer review only

References

1. World Health Organization. Novel Coronavirus (2019-nCoV): situation report, 11. Geneva: World Health Organization, 2020.
2. World Health Organization. Coronavirus disease (COVID-19): Serology, antibodies and immunity: Accessed; 2021 [Available from: https://www.who.int/news-room/questions-and-answers/item/coronavirus-disease-covid-19-serology.
3. Verity R, Okell LC, Dorigatti I, et al. Estimates of the severity of coronavirus disease 2019: a model-based analysis. *The Lancet infectious diseases* 2020;20(6):669-77.
4. World Health Organization. Population-based age-stratified seroepidemiological investigation protocol for COVID-19 virus infection, 17 March 2020: World Health Organization, 2020.
5. Bendavid E, Mulaney B, Sood N, et al. Covid-19 antibody seroprevalence in santa clara county, california. *International journal of epidemiology* 2021;50(2):410-19.
6. Havers FP, Reed C, Lim T, et al. Seroprevalence of antibodies to SARS-CoV-2 in 10 sites in the United States, March 23-May 12, 2020. *JAMA internal medicine* 2020;180(12):1576-86.
7. Montenegro P, Brotons C, Serrano J, et al. Community seroprevalence of COVID-19 in probable and possible cases at primary health care centres in Spain. *Family Practice* 2021;38(2):153-58.
8. Anna F, Goyard S, Lalanne AI, et al. High seroprevalence but short-lived immune response to SARS-CoV-2 infection in Paris. *European journal of immunology* 2021;51(1):180-90.
9. Nisar MI, Ansari N, Khalid F, et al. Serial population-based serosurveys for COVID-19 in two neighbourhoods of Karachi, Pakistan. *International Journal of Infectious Diseases* 2021;106:176-82.
10. Inbaraj LR, George CE, Chandrasingh S. Seroprevalence of COVID-19 infection in a rural district of South India: A population-based seroepidemiological study. *PloS one* 2021;16(3):e0249247.
11. Chimeddorj B, Mandakh U, Le L-V, et al. SARS-CoV-2 seroprevalence in Mongolia: Results from a national population survey. *The Lancet Regional Health-Western Pacific* 2021;17:100317.
12. Jubayer F, Kayshar S, Limon TI. First COVID-19 case in the Rohingya camp in Bangladesh: Needs proper attention. *Public Health* 2021;191:20.
13. Bhuiyan TR, Akhtar M, Akter A, et al. Seroprevalence of SARS-CoV-2 antibodies in Bangladesh related to novel coronavirus infection. *IJID Regions* 2022;2:198-203.
14. Shirin T, Bhuiyan TR, Charles RC, et al. Antibody responses after COVID-19 infection in patients who are mildly symptomatic or asymptomatic in Bangladesh. *International Journal of Infectious Diseases* 2020;101:220-25.
15. Islam MM, Khan MN, Rahman MM. Factors affecting child marriage and contraceptive use among Rohingya girls in refugee camps. *The Lancet Regional Health-Western Pacific* 2021;12:100175.
16. World Health Organization Bangladesh. Emergency: Rohingya Crisis-Monthly Situation Report # 3. https://www.who.int/bangladesh/emergencies/Rohingyacrisis/situation-report---rohingya-crisis, 2022.
17. Truelove S, Abrahim O, Altare C, et al. The potential impact of COVID-19 in refugee camps in Bangladesh and beyond: A modeling study. *PLoS medicine* 2020;17(6):e1003144.

For peer review only

18. World Health Organization Bangladesh. Emergency: Rohingya Crisis- Bi-weekly Situation Report #25. https://www.who.int/docs/default-source/searo/bangladesh/bangladesh---rohingya-crisis---pdf-reports/sitreps/2019/bi-weekly-situation-report-25---19-december-2019.pdf?sfvrsn=4241b323_2, 2019.
19. United Nations High Commissioner for Refugees (UNHCR). Joint Government of Bangladesh - UNHCR Population factsheet as of 31 August 2020. Population factsheet. https://data.unhcr.org/en/documents/details/78973: UNHCR.
20. OpenEpi: Open Source Epidemiologic Statistics for Public Health. Version 2.3.1; 2013.
21. Initiative HH. KoBoToolbox. *Retrieved November 14, 2017, from http://www kobotoolbox org* 2021
22. Lassaunière R, Frische A, Harboe ZB, et al. Evaluation of nine commercial SARS-CoV-2 immunoassays. *MedRxiv* 2020
23. KANTARO. Kantaro's SARS-CoV-2 IgG Antibody Test Kits https://kantarobio.com/antibody-test-kits2021 [2022.
24. Hakim F, Bhuiyan R, Akter MK, et al. Weighting National Survey Data in Bangladesh: Why, How and Which weight? Weighting survey data. *Bangladesh Medical Research Council Bulletin* 2022;47(2):118-26. doi: 10.3329/bmrcb.v47i2.57769
25. Rogan WJ, Gladen B. Estimating prevalence from the results of a screening test. *American journal of epidemiology* 1978;107(1):71-76.
26. Henrion MY. bootComb—an R package to derive confidence intervals for combinations of independent parameter estimates: Oxford University Press, 2021.
27. Barrie MB, Lakoh S, Kelly JD, et al. SARS-CoV-2 antibody prevalence in Sierra Leone, March 2021: a cross-sectional, nationally representative, age-stratified serosurvey. *BMJ Glob Health* 2021;6(11):e007271. doi: 10.1136/bmjgh-2021-007271
28. Ahmad AM, Shahzad K, Masood M, et al. COVID-19 seroprevalence in Pakistan: a cross-sectional study. *BMJ open* 2022;12(4):e055381. doi: 10.1136/bmjopen-2021-055381 [published Online First: 2022/04/08]
29. Levin AT, Owusu-Boaitey N, Pugh S, et al. Assessing the burden of COVID-19 in developing countries: systematic review, meta-analysis and public policy implications. *BMJ Glob Health* 2022;7(5):e008477. doi: 10.1136/bmjgh-2022-008477
30. Poletti P, Tirani M, Cereda D, et al. Association of age with likelihood of developing symptoms and critical disease among close contacts exposed to patients with confirmed SARS-CoV-2 infection in Italy. *JAMA network open* 2021;4(3):e211085-e85.
31. Bhuiyan TR, Hulse JD, Hegde ST, et al. SARS-CoV-2 seroprevalence before Delta variant surge, Chattogram, Bangladesh, March–June 2021. *Emerging infectious diseases* 2022;28(2):429.
32. Malani A, Shah D, Kang G, et al. Seroprevalence of SARS-CoV-2 in slums versus non-slums in Mumbai, India. *The Lancet Global Health* 2021;9(2):e110-e11.
33. Stringhini S, Wisniak A, Piumatti G, et al. Seroprevalence of anti-SARS-CoV-2 IgG antibodies in Geneva, Switzerland (SEROCoV-POP): a population-based study. *The Lancet* 2020;396(10247):313-19.
34. Lopez-Pena P, Davis CA, Mobarak AM, et al. Prevalence of COVID-19 symptoms, risk factors, and health behaviors in host and refugee communities in Cox's Bazar: A representative panel study. *Bulletin of the World Health Organization* 2020;11
35. Alali WQ, Bastaki H, Longenecker JC, et al. Seroprevalence of SARS-CoV-2 in migrant workers in Kuwait. *Journal of travel medicine* 2021;28(2):taaa223.
36. Barchuk A, Skougarevskiy D, Titaev K, et al. Seroprevalence of SARS-CoV-2 antibodies in Saint Petersburg, Russia: a population-based study. *Scientific reports* 2021;11(1):1-9.
37. Carrat F, de Lamballerie X, Rahib D, et al. Seroprevalence of SARS-CoV-2 among adults in three regions of France following the lockdown and associated risk factors: a multicohort study. 2020

For peer review only

38. Roederer T, Mollo B, Vincent C, et al. Seroprevalence and risk factors of exposure to COVID-19 in homeless people in Paris, France: a cross-sectional study. *The Lancet Public Health* 2021;6(4):e202-e09.
39. Rivas MN, Ebinger JE, Wu M, et al. BCG vaccination history associates with decreased SARS-CoV-2 seroprevalence across a diverse cohort of health care workers. *The Journal of clinical investigation* 2021;131(2)
40. Escobar LE, Molina-Cruz A, Barillas-Mury C. BCG vaccine protection from severe coronavirus disease 2019 (COVID-19). *Proceedings of the National Academy of Sciences* 2020;117(30):17720-26.
41. Garzon-Chavez D, Rivas-Condo J, Echeverria A, et al. COVID-19 infection and previous bcg vaccination coverage in the Ecuadorian population. *Vaccines* 2021;9(2):91.

Figure Legends:

Figure 1. Flow diagram showing selection of respondents of the FDMN population, Bangladesh December 2020

Note: FDMN, Forcibly Displaced Myanmar Nationals

*Estimated from FDMN population frame of Refugee Relief and Repatriation Commissioner (RRRC) & United Nations High Commissioner for Refuge (UNHCR)

Figure 2. Age-specific weighted test adjusted seroprevalence in male and female participants with or without history of symptoms, FDMN population, Bangladesh December 2020 (N=3446): Panel A: female, Panel B: male & Panel C: all subjects. Error bars indicate 95% confidence intervals

Note: FDMN, Forcibly Displaced Myanmar Nationals. Weighted to FDMN Population size (N=860,697) and distribution published by Refugee Relief and Repatriation Commissioner & United Nations High Commissioner for Refuge, 2020. Rogan-Gladen formula was used to adjust seroprevalence estimates to account for test sensitivity and specificity of Wantai kits validated by Kantaro[25]

Figure 3. Weighted test adjusted seroprevalence of SARS-CoV-2 among self-reported comorbid conditions, FDMN population, Bangladesh December 2020 (N=3446): Panel A: type of comorbidity, Panel B: number of comorbidities. Error bars indicate 95% confidence intervals

Note: FDMN, Forcibly Displaced Myanmar Nationals. Weighted to FDMN Population size (N=860,697) and distribution published by Refugee Relief and Repatriation Commissioner & United Nations High Commissioner for Refuge, 2020. Rogan-Gladen formula was used to adjust seroprevalence estimates to account for test sensitivity and specificity of Wantai kits validated by Kantaro[25]

For peer review only

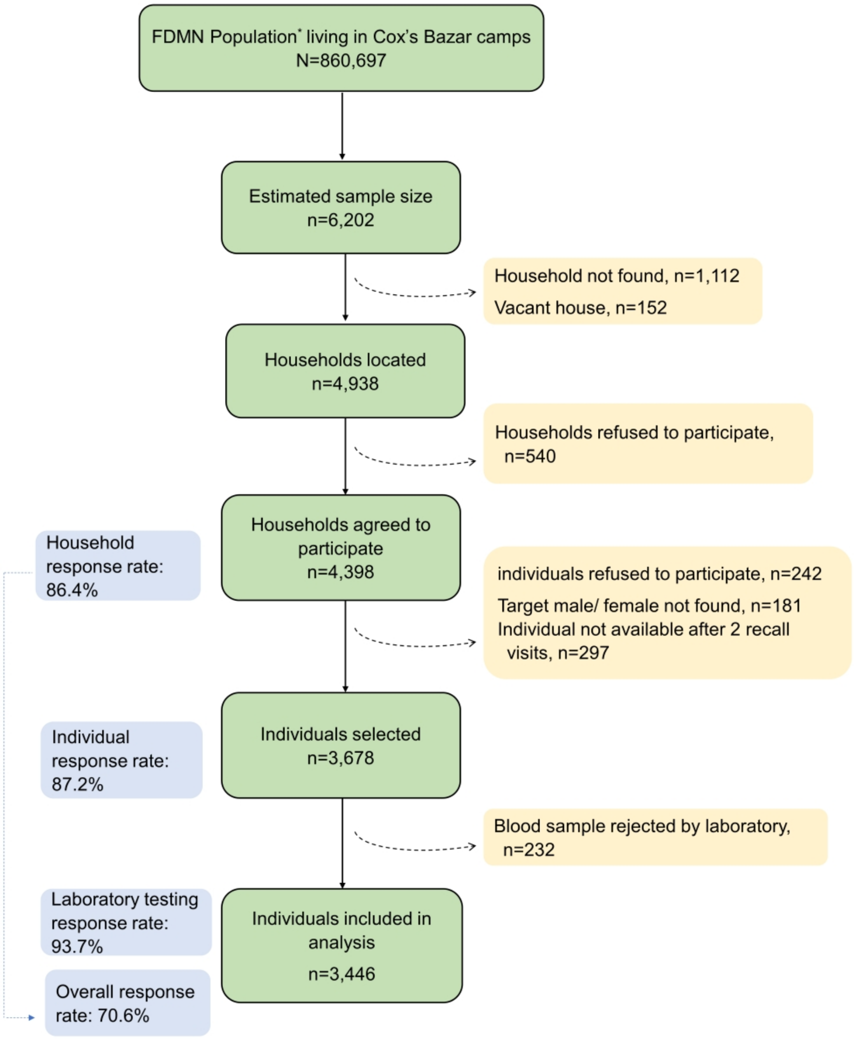

Flow diagram showing selection of respondents of the FDMN population, Bangladesh December 2020

Note: FDMN, Forcibly Displaced Myanmar Nationals
*Estimated from FDMN population frame of Refugee Relief and Repatriation Commissioner (RRRC) & United Nations High Commissioner for Refuge (UNHCR)

338x414mm (300 x 300 DPI)

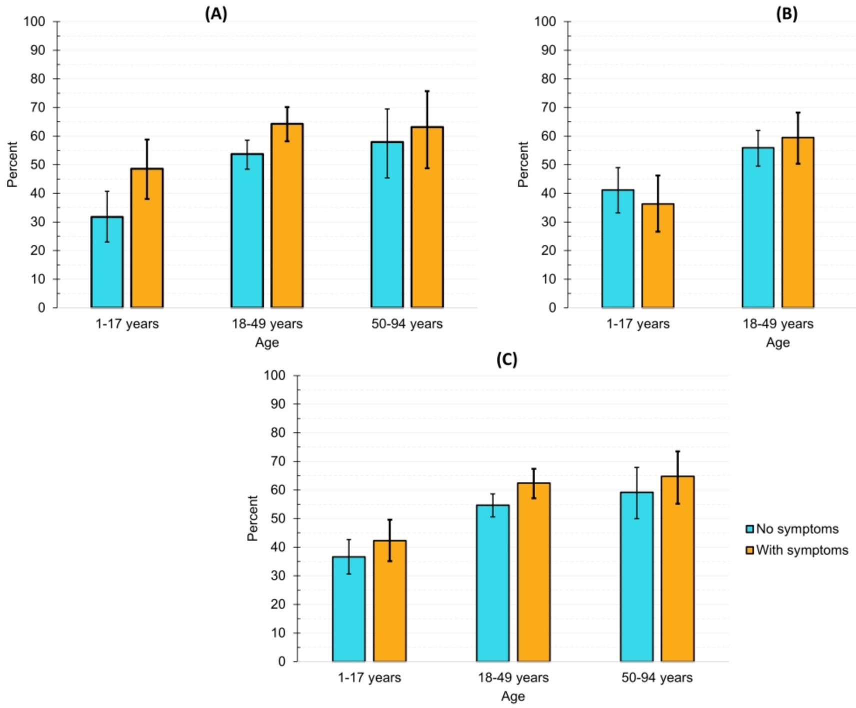

Age-specific weighted test adjusted seroprevalence in male and female participants with or without history of symptoms, FDMN population, Bangladesh December 2020 (N=3446): Panel A: female, Panel B: male & Panel C: all subjects. Error bars indicate 95% confidence intervalsNote: FDMN, Forcibly Displaced Myanmar Nationals. Weighted to FDMN Population size (N=860,697) and distribution published by Refugee Relief and Repatriation Commissioner & United Nations High Commissioner for Refuge, 2020. Rogan-Gladen formula was used to adjust seroprevalence estimates to account for test sensitivity and specificity of Wantai kits validated by Kantaro[25]

338x240mm (300 x 300 DPI)

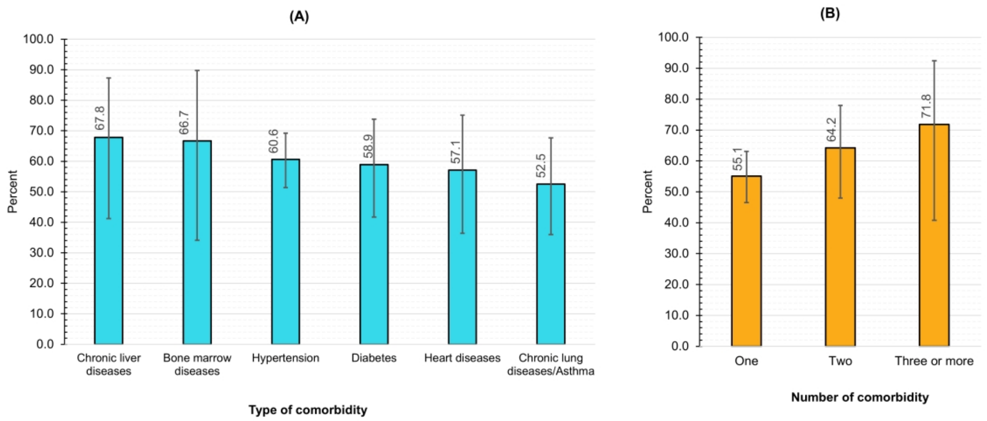

Weighted test adjusted seroprevalence of SARS-CoV-2 among self-reported comorbid conditions, FDMN population, Bangladesh December 2020 (N=3446): Panel A: type of comorbidity, Panel B: number of comorbidities. Error bars indicate 95% confidence intervals""Note: FDMN, Forcibly Displaced Myanmar Nationals. Weighted to FDMN Population size (N=860,697) and distribution published by Refugee Relief and Repatriation Commissioner & United Nations High Commissioner for Refuge, 2020. Rogan-Gladen formula was used to adjust seroprevalence estimates to account for test sensitivity and specificity of Wantai kits validated by Kantaro[25]

338x150mm (300 x 300 DPI)

Supplemental Material

Seroprevalence of SARS-CoV-2 antibodies among Forcibly Displaced Myanmar Nationals in Cox's Bazar, Bangladesh 2020: a population-based cross-sectional study

Mahbubur Rahman[1*], Samsad Rabbani Khan [1], A S M Alamgir [1], David S. Kennedy [2], Ferdous Hakim [3], Egmond Samir Evers[2], Nawroz Afreen [1], Ahmed Nawsher Alam [1], Md Sahidul Islam [3], Debashish Paul [2], Rijwan Bhuiyan [4], Raisul Islam [2], Adneen Moureen [5], M Salimuzzaman [1], Mallick Masum Billah [1], Ahmed Raihan Sharif [1], Mst. Khaleda Akter [3], Sharmin Sultana [1], Manjur Hossain Khan [1], Kai Von Harbou [2], Mohammad Mostafa Zaman [3], Tahmina Shirin [1], Meerjady Sabrina Flora [6]

[1]Institute of Epidemiology, Disease Control and Research (IEDCR), Dhaka, Bangladesh

[2]WHO Emergency Sub-Office, World Health Organization, Cox's Bazar, Bangladesh

[3]Research and Publication, World Health Organization, Dhaka, Bangladesh

[4]Coordination Center, Ministry of Health and Family Welfare, Cox's Bazar, Bangladesh

[5]IEDCR Field Laboratory, World Health Organization, Cox's Bazar, Bangladesh

[6]Directorate General of Health Services (DGHS), Dhaka, Bangladesh

Contents

Weighted and test adjusted prevalence 2

Bootstrapping for confidence interval 3

Supplementary Table 1. Responses at household, individual and laboratory testing levels, FDMN seroprevalence survey, Bangladesh December 2020 4

Supplementary Table 2: test adjusted over weighted SARS-CoV-2 antibody prevalence by sex across covariates, FDMN population, Bangladesh 2020 5

Supplementary Figure 1. FDMN camps in Ukhiya and Teknaf of Cox's Bazar district, Bangladesh December 2020 6

Supplementary Figure 2: Test adjusted over weighted seroprevalence among respondent occupation, FDMN population, Bangladesh December 2020 7

For peer review only

Weighted and test adjusted prevalence

Primarily we used WANTAI total antibody SARS-CoV-2 ELISA kits to estimate the seroprevalence, which has some limitations and could result in false positives and false negatives estimates. In order to validate the test result, we used Kantaro IgG ELISA test kit. We took the following approach to adjust the seroprevalence of WANTAI by KANTARO kits.

Let $\pi = P(K^+)$ represent the population prevalence of antibodies to SARS-CoV-2 and let $p = P(W^+)$ be the proportion of participants who test positive in WANTAI. Let, $s_e = P(W^+|K^+)$ be the sensitivity of the test (the probability of testing positive in WANTAI given an individual is positive in KANTARO), and let, $s_p = P(K^-)$ be the specificity of the test (the probability of testing negative in WANTAI given an individual is negative in KANTARO). We can write the expected fraction of positive tests, *p*, as follows:

$$p = \pi s_e + (1 - \pi)(1 - s_p)$$

Therefore, the test adjusted estimated seroprevalence is expressed using following formula [1]-

$$\pi = \frac{p - (1 - s_p)}{s_e\,(1 - s_p)} \qquad (1)$$

Of 3,446 total samples in our study, we found 2,090 positives for WANTAI. Again, we used KANTARO quantitative test to those WANTAI positive samples and we found 290 negatives. Since we didn't test any negative WANTAI samples, and we assumed that all negative samples in WANTAI are also negative in KANTARO. A confusion matrix is displayed below-

		Kantaro		
**Wantai**		p	n	Total
	p	1800	290	2090
	n	0	1356	1356
	Total	1800	1646	3046

Table: Confusion Matrix between WANTAI and KANTARO where *'p'* denoting the positive and *'n'* denoting the negative test result.

Sensitivity, $s_e = \frac{1800}{1800 + 0} = 1.00$, and specificity, $s_p = \frac{1356}{290 + 1356} = 0.8238$

Therefore, overall seroprevalence, $\pi = \frac{0.574 - (1 - 0.8238)}{1 - (1 - 0.8238)} = 0.4829$

Bootstrapping for confidence interval

We calculated confidence intervals on our weighted sample prevalence estimates based on a non-parametric highest density interval (HDI) bootstrap [2]. Our bootstrap procedure resamples data from our actual datasets for sensitivity, specificity, and prevalence. We use the following steps-

- First, we draw a single bootstrap sample (i.e. with replacement) from the beta distribution of the sensitivity data. For this sample, we calculate the mean value of sensitivity. Let s_{e_j}represent the value for the *jth* iteration of this procedure.
- First, we draw a single bootstrap sample (i.e. with replacement) from the beta distribution of the specificity data. For this sample, we calculate the mean value of specificity. Let s_{p_j}represent the value for the *jth* iteration of this procedure.
- For our weighted estimate, we calculate weighted prevalence of WANTAI in the bootstrap sample . Let p_j represent this value for the jth iteration of this procedure.
- We then calculate the test adjusted prevalence of antibodies to SARS-CoV-2 for the *jth* bootstrap sample using the equation (1)
- Repeat the steps above for j=1...1,000,000 bootstrap samples.
- In our tables, we report the highest density interval (HDI) of the distributions of π_j over the 1,000,000 bootstrap samples as the lower and upper ends of the 95% bootstrap confidence intervals.

The confidence intervals for the test-adjusted estimates were derived using bootstrap sampling, with 1,000,000 parametric bootstrap samples for each estimate, using the "*adjPrevSensSpecCI*" function of the "*bootComb*" R package.

References

1. Chimeddorj B, Mandakh U, Le LV, Bayartsogt B, Deleg Z, Enebish O, Altanbayar O, Magvan B, Gantumur A, Byambaa O, Enebish G. SARS-CoV-2 seroprevalence in Mongolia: Results from a national population survey. The Lancet Regional Health-Western Pacific. 2021 Dec 1;17:100317.
2. Bendavid E, Mulaney B, Sood N, Shah S, Bromley-Dulfano R, Lai C, Weissberg Z, Saavedra-Walker R, Tedrow J, Bogan A, Kupiec T. Covid-19 antibody seroprevalence in santa clara county, california. International journal of epidemiology. 2021 Apr;50(2):410-9.
3. Henrion MY. bootComb—an R package to derive confidence intervals for combinations of independent parameter estimates.

For peer review only

Supplemental Table 1. Responses at household, individual and laboratory testing levels, FDMN seroprevalence survey, Bangladesh December 2020

Responses	All	
	n	%
At household level		
Roster completed (RC)	4398	70.9
Vacant house/ No HH respondent available (VH)	152	2.5
House not found*	1112	17.9
Refused interview (RefI)	540	8.7
Total	6202	100
Household response rate (HRR)†		86.4
At individual level		
Completed (C)	3678	83.6
No one eligible*	181	4.1
Unavailable (U)	297	6.8
Refused (R)	242	5.5
Total	4398	100
Individual response rate (IRR)‡		87.2
At laboratory testing level		
Laboratory testing completed (C)	3446	93.7
Sample rejected by laboratory (Rej)	232	6.3
Total	3678	100
Laboratory testing response rate (LTRR)§		93.7
Overall response rate¶		70.6

Note: FDMN, Forcibly Displaced Myanmar Nationals
*Not included as sample as they do not qualify[1] as sample for the Survey
†Household response rate (%)=[RCx100]/[RC+VH+RefI]

‡Individual response rate (%)=[Cx100]/[C+II+U+R+RI]

§Laboratory testing response rate (%)=[Cx100]/[C+Rej]

¶Overall response rate (%)=HRR*IRR*LTRR/(100*100)

[1]The American Association for Public Opinion Research. 2016. Standard Definitions: Final Dispositions of Case Codes and Outcome Rates for Surveys. 9th edition. AAPOR. Available from: https://www.aapor.org/AAPOR_Main/media/publications/Standard-Definitions20169theditionfinal.pdf (Accessed on 3 September 2019)

For peer review only

Supplemental Table 2: test adjusted over weighted SARS-CoV-2 antibody prevalence by sex across covariates, FDMN population, Bangladesh 2020

Variable	Weighted prevalence, % (95% CI)			Weighted & kit adjusted prevalence, % (95% CI)		
	Overall	Female	Male	Overall	Female	Male
Age, years						
All	57.4 (55.1-59.7)	57.4 (54.2-60.6)	57.4 (54.2-60.6)	48.3 (45.3-51.4)	48.3 (44.2-52.3)	48.3 (44.2-52.3)
1-17	49.4 (45.6-53.2)	48.6 (43.0-54.3)	50.2 (45.1-55.2)	38.6 (33.8-43.4)	37.6 (30.7-44.7)	39.5 (33.2-45.8)
18-94	65.5 (63.2-67.8)*	65.4 (62.4-68.2)*	65.7 (62.0-69.3)*	58.1 (55.2-61.1)*	58.0 (54.3-61.6)*	58.4 (53.8-62.9)*
18-49	64.9 (62.3-67.4)	65.1 (61.9-68.1)	64.7 (60.4-68.8)	57.4 (54.1-60.6)	57.6 (53.6-61.4)	57.1 (51.9-62.3)
50-94	68.5 (63.0-73.5)*	67.2 (59.5-74.2)*	69.5 (61.7-76.3)*	61.8 (55.1-68.0)*	60.2 (50.9-68.9)*	63.0 (53.7-71.4)*
Education† (n=3,160)						
No formal education	61.7 (58.8-64.5)	61.4 (57.9-64.8)	62.1 (57.0-67.0)	53.5 (49.8-57.1)	53.1 (48.8-57.4)	54.0 (47.7-60.1)
Primary above	61.8 (58.3-65.2)	59.0 (53.0-64.8)	63.8 (59.5-67.9)	53.6 (49.2-57.9)	50.2 (42.9-57.4)	56.1 (50.8-61.1)
Camp location						
Teknaf	54.8 (48.9-60.6)	59.7 (51.0-67.7)	49.4 (41.3-57.6)	45.1 (37.8-52.2)	51.1 (40.6-60.9)	38.6 (28.7-48.7)
Ukhiya	58.0 (55.5-60.4)	56.9 (53.5-60.3)	59.1 (55.5-62.5)*	49.0 (45.8-52.2)	47.7 (43.3-51.9)	50.4 (45.8-54.6)*
Smoking‡ (n=2,392)						
No	67.5 (64.9-70.0)	65.3 (62.2-68.2)	71.8 (67.1-76.1)	60.5 (57.3-63.7)	57.9 (54.0-61.5)	65.8 (60.1-71.2)
Yes	59.1 (53.9-64.2)*	66.5 (55.6-75.9)	57.5 (51.6-63.2)*	50.4 (43.9-56.6)*	59.3 (46.5-71.2)	48.4 (41.2-55.5)*
BCG vaccination						
No	58.8 (52.7-64.7)	58.9 (50.0-67.2)	58.7 (50.2-66.7)	50.0 (42.5-57.3)	50.1 (39.4-60.4)	49.9 (39.5-59.7)
Yes	57.2 (54.7-59.6)	57.2 (53.8-60.6)	57.2 (53.7-60.6)	48.0 (44.8-51.2)	48.0 (43.8-52.4)	48.0 (43.6-52.3)
Any relevant symptoms						
No symptom	55.3 (52.3-58.2)	53.3 (49.1-57.4)	57.3 (53.2-61.4)	45.7 (41.9-49.5)	43.3 (38.1-48.5)	48.2 (43.1-53.3)
Any symptom	61.2 (57.7-64.6)*	64.6 (59.9-69.1)*	57.5 (52.3-62.5)	52.9 (48.5-57.2)*	57.0 (51.2-62.6)*	48.4 (42.0-54.6)
Any comorbidity						
No comorbidity	56.5 (54.0-58.9)	56.1 (52.6-59.5)	56.9 (53.5-60.2)	47.2 (43.9-50.4)	46.7 (42.3-51.0)	47.7 (43.4-51.9)
Any comorbidity	65.2 (59.2-70.7)*	67.3 (59.8-74.0)*	62.4 (52.4-71.4)	57.8 (50.4-64.5)*	60.3 (51.4-68.7)*	54.4 (42.4-65.5)

Note: FDMN, Forcibly Displaced Myanmar Nationals
*P<0.05
†Among those aged 6 years or more
‡Among those aged 18 years or more

For peer review only

For peer review only

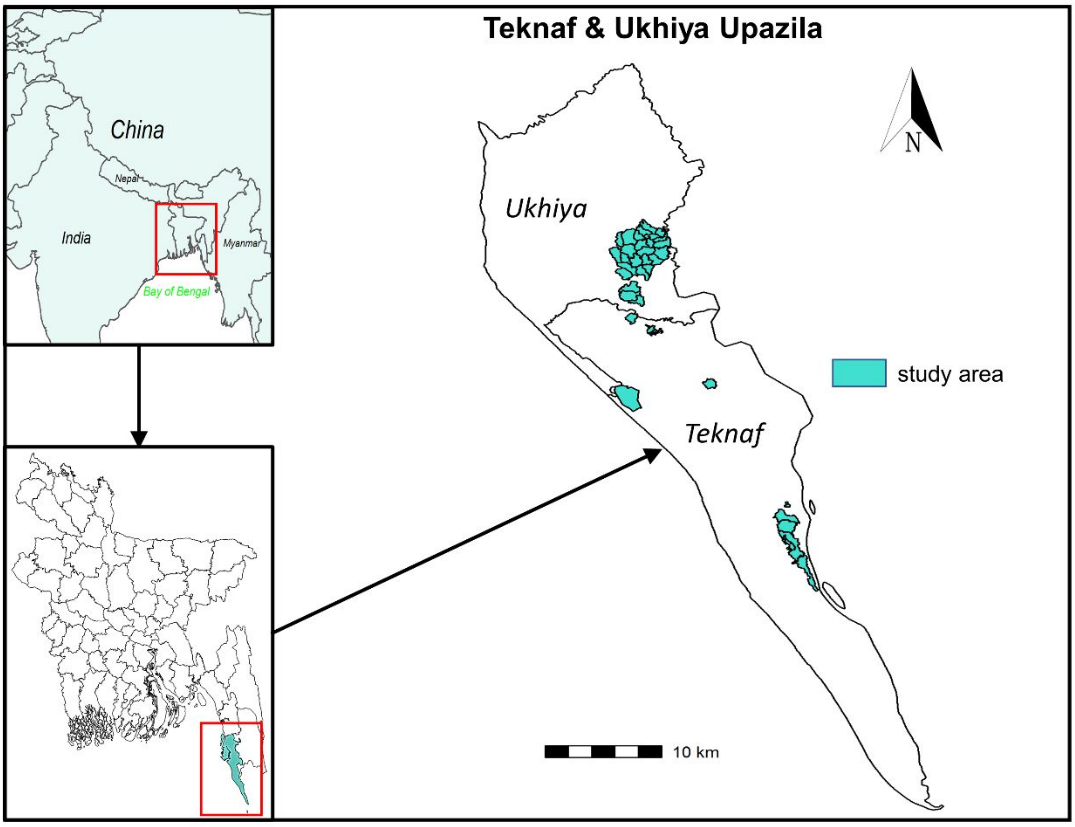

Supplemental Figure 1. FDMN camps in Ukhiya and Teknaf of Cox's Bazar district, Bangladesh December 2020

Note: FDMN, Forcibly Displaced Myanmar Nationals

review only

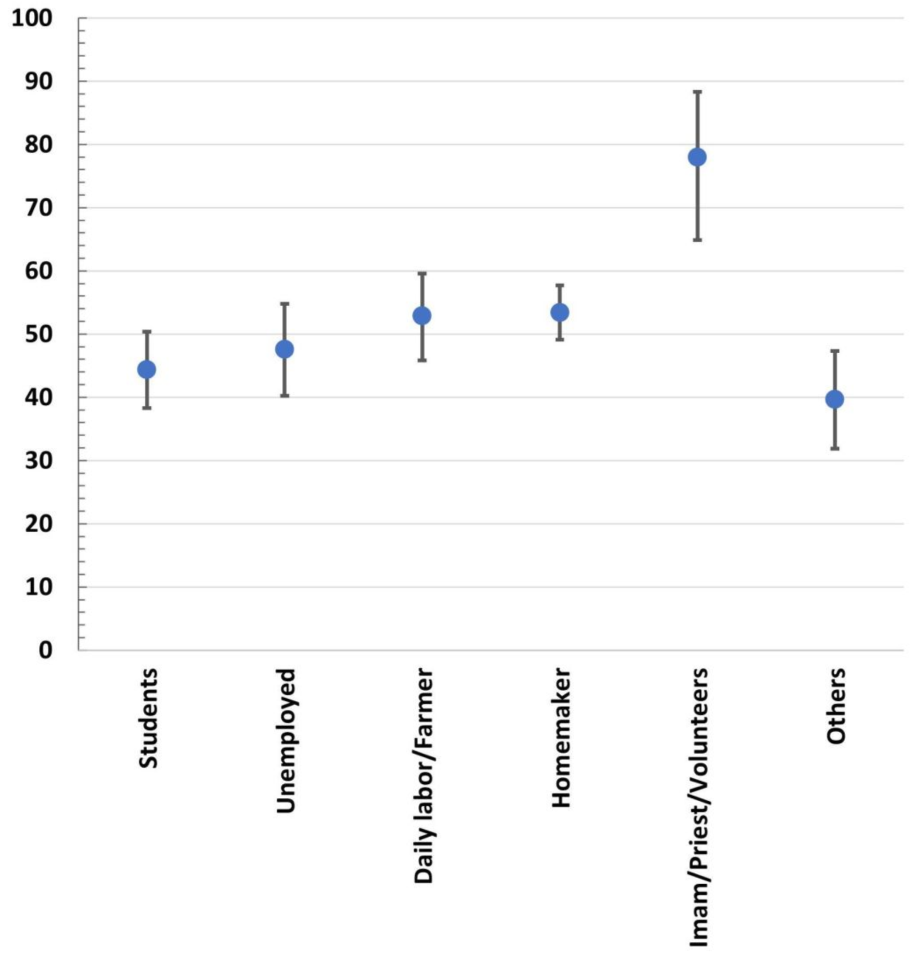

Supplemental Figure 2: Test adjusted over weighted seroprevalence among respondent occupation, FDMN population, Bangladesh December 2020

Note: Blue dot represents the prevalence and error bar indicating confidence interval.

STROBE Statement—Checklist of items that should be included in reports of ***cross-sectional studies***

	Item No	Recommendation	Page No
**Title and abstract**	1	(*a*) Indicate the study's design with a commonly used term in the title or the abstract	3
		(*b*) Provide in the abstract an informative and balanced summary of what was done and what was found	4
**Introduction**			
Background/rationale	2	Explain the scientific background and rationale for the investigation being reported	6
Objectives	3	State specific objectives, including any prespecified hypotheses	6
**Methods**			
Study design	4	Present key elements of study design early in the paper	7
Setting	5	Describe the setting, locations, and relevant dates, including periods of recruitment, exposure, follow-up, and data collection	7
Participants	6	(*a*) Give the eligibility criteria, and the sources and methods of selection of participants	7
Variables	7	Clearly define all outcomes, exposures, predictors, potential confounders, and effect modifiers. Give diagnostic criteria, if applicable	7
Data sources/ measurement	8*	For each variable of interest, give sources of data and details of methods of assessment (measurement). Describe comparability of assessment methods if there is more than one group	8
Bias	9	Describe any efforts to address potential sources of bias	9
Study size	10	Explain how the study size was arrived at	7
Quantitative variables	11	Explain how quantitative variables were handled in the analyses. If applicable, describe which groupings were chosen and why	9
Statistical methods	12	(*a*) Describe all statistical methods, including those used to control for confounding	9
		(*b*) Describe any methods used to examine subgroups and interactions	
		(*c*) Explain how missing data were addressed	
		(*d*) If applicable, describe analytical methods taking account of sampling strategy	10
		(*e*) Describe any sensitivity analyses	
**Results**			
Participants	13*	(a) Report numbers of individuals at each stage of study—eg numbers potentially eligible, examined for eligibility, confirmed eligible, included in the study, completing follow-up, and analysed	10
		(b) Give reasons for non-participation at each stage	10
		(c) Consider use of a flow diagram	21
Descriptive data	14*	(a) Give characteristics of study participants (eg demographic, clinical, social) and information on exposures and potential confounders	10
		(b) Indicate number of participants with missing data for each variable of interest	11
Outcome data	15*	Report numbers of outcome events or summary measures	12
Main results	16	(*a*) Give unadjusted estimates and, if applicable, confounder-adjusted estimates and their precision (eg, 95% confidence interval). Make clear which confounders were adjusted for and why they were included	12

For peer review only

		(*b*) Report category boundaries when continuous variables were categorized	12
		(*c*) If relevant, consider translating estimates of relative risk into absolute risk for a meaningful time period	
Other analyses	17	Report other analyses done—eg analyses of subgroups and interactions, and sensitivity analyses	13
**Discussion**			
Key results	18	Summarise key results with reference to study objectives	14
Limitations	19	Discuss limitations of the study, taking into account sources of potential bias or imprecision. Discuss both direction and magnitude of any potential bias	16
Interpretation	20	Give a cautious overall interpretation of results considering objectives, limitations, multiplicity of analyses, results from similar studies, and other relevant evidence	16-17
Generalisability	21	Discuss the generalisability (external validity) of the study results	16
**Other information**			
Funding	22	Give the source of funding and the role of the funders for the present study and, if applicable, for the original study on which the present article is based	17

*Give information separately for exposed and unexposed groups.

Note: An Explanation and Elaboration article discusses each checklist item and gives methodological background and published examples of transparent reporting. The STROBE checklist is best used in conjunction with this article (freely available on the Web sites of PLoS Medicine at http://www.plosmedicine.org/, Annals of Internal Medicine at http://www.annals.org/, and Epidemiology at http://www.epidem.com/). Information on the STROBE Initiative is available at www.strobe-statement.org.

For peer review only

BMJ Open

Seroprevalence of SARS-CoV-2 antibodies among Forcibly Displaced Myanmar Nationals in Cox's Bazar, Bangladesh 2020: a population-based cross-sectional study

Journal:	*BMJ Open*
Manuscript ID	bmjopen-2022-066653.R1
Article Type:	Original research
Date Submitted by the Author:	24-Oct-2022
Complete List of Authors:	Rahman , Mahbubur ; Institute of Epidemiology Disease Control and Research, Epidemiology<br>Khan, Samsad; Institute of Epidemiology Disease Control and Research, Epidemiology<br>Alamgir, A. S. M; Institute of Epidemiology Disease Control and Research, Virology<br>Kennedy , David ; World Health Organization, WHO Emergency Sub-Office<br>Hakim, Ferdous; World Health Organization Bangladesh, Research and Publication<br>Evers, Egmond; World Health Organization, WHO Emergency Sub-Office<br>Afreen, Nawroz ; Institute of Epidemiology Disease Control and Research, Epidemiology<br>Alam, Ahmed; Institute of Epidemiology Disease Control and Research, Virology<br>Islam, Md. Sahidul; World Health Organization Bangladesh, Research & Publications<br>Paul, Debashish; World Health Organization, WHO Emergency Sub-Office<br>Bhuiyan, Rijwan; Ministry of Health and Family Welfare, Co-ordination Center<br>Islam, Raisul; World Health Organization, WHO Emergency Sub-Office<br>Moureen, Adneen ; World Health Organization, IEDCR Field Laboratory<br>Salimuzzaman , M; Institute of Epidemiology Disease Control and Research<br>Billah, Mallick; Institute of Epidemiology Disease Control and Research, Epidemiology<br>Sharif , Ahmed ; Institute of Epidemiology Disease Control and Research, Epidemiology<br>Akter, Mst. Khaleda; World Health Organization Bangladesh, Research and Publication<br>Sultana , Sharmin; Institute of Epidemiology Disease Control and Research, Virology<br>Khan , Manjur; Institute of Epidemiology Disease Control and Research, Virology<br>von Harbou, Kai; World Health Organization, WHO Emergency Sub-Office<br>Zaman, MM; World Health Organization, Research and Publication<br>Shirin, Tahmina; Institute of Epidemiology Disease Control and Research, Director<br>Flora, Meerjady Sabrina ; Directorate General of Health Services ,

	Additional Director General
**Primary Subject Heading**:	Epidemiology
Secondary Subject Heading:	Infectious diseases, Public health
Keywords:	COVID-19, Epidemiology < INFECTIOUS DISEASES, Public health < INFECTIOUS DISEASES, EPIDEMIOLOGY, PUBLIC HEALTH

SCHOLARONE™
Manuscripts

I, the Submitting Author has the right to grant and does grant on behalf of all authors of the Work (as defined in the below author licence), an exclusive licence and/or a non-exclusive licence for contributions from authors who are: i) UK Crown employees; ii) where BMJ has agreed a CC-BY licence shall apply, and/or iii) in accordance with the terms applicable for US Federal Government officers or employees acting as part of their official duties; on a worldwide, perpetual, irrevocable, royalty-free basis to BMJ Publishing Group Ltd ("BMJ") its licensees and where the relevant Journal is co-owned by BMJ to the co-owners of the Journal, to publish the Work in this journal and any other BMJ products and to exploit all rights, as set out in our licence.

The Submitting Author accepts and understands that any supply made under these terms is made by BMJ to the Submitting Author unless you are acting as an employee on behalf of your employer or a postgraduate student of an affiliated institution which is paying any applicable article publishing charge ("APC") for Open Access articles. Where the Submitting Author wishes to make the Work available on an Open Access basis (and intends to pay the relevant APC), the terms of reuse of such Open Access shall be governed by a Creative Commons licence – details of these licences and which Creative Commons licence will apply to this Work are set out in our licence referred to above.

Other than as permitted in any relevant BMJ Author's Self Archiving Policies, I confirm this Work has not been accepted for publication elsewhere, is not being considered for publication elsewhere and does not duplicate material already published. I confirm all authors consent to publication of this Work and authorise the granting of this licence.

For peer review only

Seroprevalence of SARS-CoV-2 antibodies among Forcibly Displaced Myanmar Nationals in Cox's Bazar, Bangladesh 2020: a population-based cross-sectional study

Short title: Seroprevalence of SARS-CoV-2 in FDMNs

Authors' list:

Mahbubur Rahman[1*], Samsad Rabbani Khan [1], A S M Alamgir [1], David S. Kennedy [2], Ferdous Hakim [3], Egmond Samir Evers[2], Nawroz Afreen [1], Ahmed Nawsher Alam [1], Md Sahidul Islam [3], Debashish Paul [2], Rijwan Bhuiyan [4], Raisul Islam [2], Adneen Moureen [5], M Salimuzzaman [1], Mallick Masum Billah [1], Ahmed Raihan Sharif [1], Mst. Khaleda Akter [3], Sharmin Sultana [1], Manjur Hossain Khan [1], Kai Von Harbou [2], Mohammad Mostafa Zaman [3], Tahmina Shirin [1], Meerjady Sabrina Flora [6]

Authors' affiliation:

[1]Institute of Epidemiology, Disease Control and Research (IEDCR), Dhaka, Bangladesh

[2]WHO Emergency Sub-Office, World Health Organization, Cox's Bazar, Bangladesh

[3]Research and Publication, World Health Organization, Dhaka, Bangladesh

[4]Coordination Center, Ministry of Health and Family Welfare, Cox's Bazar, Bangladesh

[5]IEDCR Field Laboratory, World Health Organization, Cox's Bazar, Bangladesh

[6]Directorate General of Health Services (DGHS), Dhaka, Bangladesh

*Corresponding author: Mahbubur Rahman

dr_mahbub@yahoo.com

Institute of Epidemiology, Disease Control and Research (IEDCR),

Mohakhali, Dhaka - 1212

Bangladesh

Word Count (excluding title page, abstract, references, figures, tables): 3904

For peer review only

Abstract

Objectives:

The study aimed to determine the seroprevalence, the fraction of asymptomatic infections, and risk factors of SARS-CoV-2 infections among the Forcibly Displaced Myanmar Nationals (FDMNs).

Design:

It was a population-based two-stage cross-sectional study at the level of households.

Setting:

The study was conducted in December 2020 among household members of the FDMN population living in the 34 camps of Ukhia and Teknaf Upazila of Cox's Bazar district in Bangladesh.

Participants:

Among 860,697 FDMNs residing in 187,517 households, 3,446 were recruited for the study. One individual aged one year or older was randomly selected from each targeted household.

Primary and secondary outcome measures:

Blood samples from respondents were tested for total antibodies for SARS-CoV-2 using Wantai ELISA kits, and later positive samples were validated by Kantaro kits.

Results:

More than half (55.3%) of the respondents were females, aged 23 median (interquartile range, 14–35) years and more than half (58.4%) had no formal education. Overall, 2,090 of 3,446 study participants tested positive for SARS-CoV-2 antibody. The weighted and test adjusted seroprevalence (95% confidence interval) was 48.3% (45.3–51.4%) which did not differ by the sexes. Children (aged 1–17 years) had a significantly lower seroprevalence 38.6% (33.8–43.4%) compared to adults (58.1%, 55.2–61.1%). Almost half (45.7%, 41.9–49.5%) of seropositive individuals reported no relevant symptoms since March 2020. Antibody seroprevalence was higher in those with any comorbidity (57.8%, 50.4–64.5%) than those without (47.2%, 43.9–50.4%). Multivariate logistic regression analysis of all subjects identified increasing age and education as risk factors for seropositivity. In children (≤17 years), only age was significantly associated with the infection.

For peer review only

Conclusions:
In December 2020, about half of the FDMNs had antibodies against SARS-CoV-2, including those who reported no history of symptoms. Periodic serosurveys are necessary to recommend appropriate public health measures to limit transmission.

Keywords: COVID-19, Seroprevalence, FDMN, Bangladesh, Pandemic, SARS-CoV-2, Serology, Antibody

For peer review only

Strength and limitations of the study

- This study is one of the largest serosurveys in COVID-19 pandemic among displaced/refugee population.
- Two-stage population-based design of the study with households identified randomly from the Family Counting Number (FCN) using probability proportionate to size of the camps and participants recruited randomly from each selected households with a balanced number of males and females, ensured generalizability and gender equity in data collection.
- Seroprevalence estimates were adjusted to account for test sensitivity and specificity of Wantai test kit validated by Kantaro to address issue of cross-reactivity.
- The household roster could not be completed for all of targeted subjects because the listing of households was done several months before the survey. Non-response at the household level was higher than expected. This might have caused overestimation of the seroprevalence, which was addressed by population and household level weighting.
- The study outcome relied on testing of antibody response, estimates of which might be affected by the waning antibody levels over time and the Wantai ELISA might have cross-reactivity with antibodies to other common human coronaviruses leading to a false-positive result in some instances.

For peer review only

Introduction

A novel pathogen's epidemiological and serological characteristics at the beginning of a pandemic remain uncertain. After the initial detection of severe acute respiratory syndrome coronavirus 2 (SARS-CoV-2) in Wuhan of China in December 2019,[1] surveillance has focused primarily on clinical presentations and severity of COVID-19 disease. A full spectrum of the disease was not evident[2 3] Real-time reverse transcriptase-polymerase chain reaction (rRT-PCR) testing of people can identify patients, including those with mild symptoms. However, previous SARS-CoV-2 infections can be identified using antibody tests, including those who recovered or were not tested being asymptomatic.[4]

The global and regional data on the seroprevalence of antibodies against SARS-CoV-2 ranged during 2020 from 1.4% to 26.0% in countries like the USA,[5 6] Spain,[7] France,[8] Pakistan,[9] India,[10] and Mongolia.[11] The first case of COVID-19 was reported in the Bangladeshi population on 8 March 2020, and the first case in Forcibly Displaced Myanmar Nationals (FDMNs) in Cox's Bazar district on 15 May 2020.[12] One study among Bangladeshi population between April and October 2020[13] estimated the national seroprevalence of immunoglobulins G (IgG) and M (IgM) to be 30.4% and 39.7%, respectively. In Dhaka, the seroprevalence of IgG was 35.4% in non-slum areas and 63.5% in slum areas. In areas outside of Dhaka, the seroprevalence of IgG was 37.5% in urban areas and 28.7% in rural areas. Another study in Bangladesh reported a considerable variation of antibodies in symptomatic and asymptomatic COVID-19 patients.[14] However, seroprevalence estimates in FDMNs are absent, and studies documenting the seroprevalence of SARS-CoV-2 antibodies among displaced populations are limited globally. Bangladesh currently hosts around one million FDMNs (commonly known as 'Rohingyas', an ethnic group in the Rakhine State of Myanmar who are forcibly displaced in Bangladesh)[15] in two sub-districts of a south-eastern frontier district, Cox's Bazar, of Bangladesh.[16]

As of 31 March 2022, 5,902 COVID-19 confirmed cases were reported from the FDMN camps with 42 deaths.[17] These camps are at high risk for COVID-19 due to their very high population density, limited access to improved sanitation , and difficulties in implementing infection control measures.[18] Several outbreaks of communicable diseases have occurred in these camps in the past,[19] including measles, diphtheria and acute watery diarrhoea.

The aim of this survey, which has been adapted from the World Health Organization (WHO) Unity Study Protocol,[4] was to estimate the seroprevalence of SARS-CoV-2

For peer review only

antibodies and the proportion of asymptomatic infections among the people living in the FDMN camps in Cox's Bazar district of Bangladesh.

Methods

Setting

We conducted this population-based two-stage cross-sectional survey in December 2020. The study was done on household members of the FDMN population using an adapted version of the WHO Unity Study Protocol.[4] All 34 FDMN camps of Cox's Bazar district were included (**Supplemental Figure 1**), which present with a population density of 40,000 people per square kilometre on average with some places reaching 70,000.[20] The sampling frame consisted of 860,697 FDMNs residing in 187,517 households registered with the Refugee Relief and Repatriation Commissioner (RRRC)-United Nations High Commissioner for Refugees (UNHCR) database as part of the RRRC-UNHCR Family Counting Exercise (FCE). UNHCR updates its FDMN database periodically collaboratively with the Government of Bangladesh.[21]

The sample size was calculated using an online tool for calculating proportion (OpenEpi).[22] Considering a hypothesized seroprevalence of 2.8%,[5] confidence limits ±0.6%, design effect (1.5) and non-response adjustment (30%), the estimated minimum sample size was 6,202.

In each camp, households were identified randomly by the Family Counting Number (FCN) using probability proportionate to size. Each identified household was assigned either as male or as female to ensure a balanced sample of males and females. One individual aged one year or more was selected from each identified household using a random number generator. Informed written consent was collected from the recruited respondents or their parents or legal guardians in case of children. Written assent was obtained from children aged 12-17 years. People who had any contraindication to venepuncture, such as bruising, swelling, infection, or broken skin, were excluded from the study (**Figure 1**).

Questionnaire and data collection tool

The questionnaire was based on the WHO Unity Protocol[4] and included questions on demographic characteristics and symptom history. The questionnaire was translated into Bangla and the common language used in FDMN camps (Burmese) for participants' better understanding. Pretesting of the questions was carried out and the questionnaire was programmed using KoBo Toolbox[23] running on Android phones and tablets. Phones and

For peer review only

tablets were password-protected, and collected data was transferred at the end of each day of the activity.

Field teams and training

The field teams consisted of doctors (team lead), nurses, laboratory technologists, paramedics, and enumerators who were proficient in the local dialect. Forty-nine teams were deployed to collect data. Each field team comprised of two data collectors and one enumerator of the same sex. Female teams collected samples from households assigned as female and male teams collected samples from households assigned as male. In addition, community health workers/ community leaders (M*ajhis)* provided support to the teams to locate the selected households. The field teams' training included interactive discussions on all questions, mock interviews, and blood sample collection exercises.

Campaign to sensitize

Sensitization meetings in all 34 FDMN camps were arranged before initiating data collection by the Camp In-charge assigned by the Government. Support from local religious leaders (e.g., imam), M*ajhis*, site management staff, and community health workers of relevant partner organisations were sought to conduct the study. A flipchart developed by WHO was used to aid these sensitization meetings.

Specimen collection, preservation, and testing procedures

Five milliliters (three ml for young children) of venous blood was collected with aseptic precautions from each of the respondents. The samples were transported to specimen collection hubs maintaining proper labelling and temperature (2-8°C). At the hubs (one for each Upazila), the samples were checked for hemolysis, lipemia and inadequate quantity and sorted and sent to the IEDCR field laboratory at Cox's Bazar Medical College, Cox's Bazar for centrifugation at 3,000 rpm for 20 min and labelled appropriately in aliquots (3 for each individual) for preservation at -80°C.

Samples with laboratory identification numbers were screened for the presence of SARS-CoV-2 virus-specific antibodies using Wantai SARS-CoV-2 Antibody ELISA kit[24] according to the manufacturer's instructions. One of the three aliquots was used for ELISA, and the remaining aliquots were stored at -80^{0}C for further testing and quality control.

The blood samples were tested for the qualitative detection of total antibodies (IgG and IgM) to SARS-CoV-2 using a two-step incubation antigen "sandwich" enzyme immunoassay where the receptor-binding domain of SARS-CoV-2 spike protein was present. The patient's serum specimen was added during the first incubation, and a second recombinant SARS-CoV-2

For peer review only

antigen was added during the second incubation, respectively. The microwells were then washed to remove unbound conjugate, and chromogen solutions were added to identify the amount of antibody captured inside the well. Wells containing specimens negative for SARS-CoV-2 antibodies remained colorless.

The positive test results were validated by Kantaro test kits[25] which is a two-phase ELISA optimized for accurate detection of SARS-CoV-2 IgG antibodies, present in human serum samples with a specificity of 99.8% and a sensitivity of 97.8%.

Quality assurance measures

The data collection procedure and teams were supervised by trained physicians. Independent monitoring visits were made by the investigators and other officials familiar with the study protocol. For external validation and quality control, 5% of the blood samples (positive and negative) were retested using Wantai kits at the IEDCR laboratory in Dhaka, the reference laboratory for SARS-CoV-2 in Bangladesh. Again, positive test results for antibodies to SARS-CoV-2 by Wantai test kits were validated by Kantaro test kits at the IEDCR reference laboratory.

Data weighting and analysis

Data were downloaded from the cloud and merged with laboratory test results with unique identification numbers of respondents. After cleaning the dataset, weighting for FDMN population[21] was done following the method described by Hakim et al.[26] Base weight was calculated using a probability of selection of respondents among the eligible number of members of the household in a cluster defined by 34 camps, five age groups, and two sexes. Later, base weight was adjusted with non-response weights separately for males and females. The final weight was generated after calibration to frame the population[21] in domains by camp, sex, and age groups. Analysis was done using the final weights.

Categorical variables were reported as numbers and percentages, and continuous variables by median (interquartile range) or mean (standard deviation) as appropriate. Chi-square was used to test the independence between seropositive and categorical factors.

Our estimation of the seroprevalence of SARS-CoV-2 proceeded in two steps. First, we estimated the weighted seroprevalence of SARS-CoV-2 to represent the FDMN population. Later we used the Rogan-Gladen formula to adjust seroprevalence estimates to account for test sensitivity and specificity of Wantai validated by Kantaro.[27] The 95% confidence intervals for the test-adjusted estimates were derived using bootstrap sampling, with

For peer review only

1,000,000 parametric bootstrap samples for each estimate, using the "adjPrevSensSpecCI" function of the "bootComb" R package.[28] More details on our bootstrap procedures are available in **Supplemental Material**.

Odds ratios (ORs) and their 95% confidence intervals were obtained using logistic regression analysis to identify the factors associated with weighted test adjusted seroprevalence. Multiple logistic regression models were applied on weighted data for children (1-17 years), adults (18 years and above), and all subjects combined. All variables were entered simultaneously into the multiple regression models. However, smoking could be used for adults only because smoking data were not collected for children. Alpha was set at 5% for considering statistical significance. All analyses were conducted using IBM SPSS version 26.0 and R version 4.1.1.

Patient and Public Involvement

Sensitization meetings were conducted in all FDMN camps before initiating data collection to ensure public involvement. Local religious leaders (e.g., imam) and M*ajhis* were involved to implement the study, especially for locating the households.
Patients or the public were not involved in the study design, or reporting, or dissemination plans.

Results

Among the estimated sample size of 6,202, the study teams were successful in locating 4,398 households yielding a response rate of 86.4%. Among the selected households, 3,678 respondents (response rate, 87.2%) completed the individual interview and provided blood samples. Among the collected blood samples, 232 were rejected in the laboratory. Therefore, blood samples from 3,446 respondents completed the laboratory test leading to a response rate of 93.7%. Data from these 3,446 respondents with completed laboratory tests were used for the final analysis. Based on these three response rates, the overall response rate was 70.6%. (**Supplemental Table 1**). All results presented are based on weighted data.

Background information of the study subjects

More than half of the respondents (55.3%) were female. More than two-thirds (69.4%) of them were aged 18 years or above. The median age (interquartile range) of all participants was 23 (14-35) years, which was not different by the sexes. More than half of the school-eligible (age ≥6 years) population reported no formal schooling (58.4%). Most of the respondents reported receiving BCG vaccination (86.3%). Nearly half (42.8%) of adult males (18 years and above) reported smoking tobacco, compared to 7.2% of females. About one in

For peer review only - http://bmjopen.bmj.com/site/about/guidelines.xhtml

eight (12.5%) reported at least one comorbid condition. More than one-third (37.0%) of the respondents reported having at least one relevant symptom of COVID-19 disease since March 2020. The symptoms included fever, cough, dyspnoea, anosmia, chills, fatigue, sore throat, headache, ageusia, nausea, vomiting, diarrhoea, wheezing, chest pain, rhinorrhoea, conjunctivitis, other respiratory symptoms, muscle aches, myalgia, rash, loss of appetite, nosebleed, and seizures (**Table 1**).

Table 1: Sociodemographic and health related background information of study participants, FDMN population, Bangladesh December 2020 (N=3446)

Variable	Overall	Female	Male
	n (%)	*n (%)*	*n (%)*
Age, years			
All	3446 (100.0)	1906 (100.0)	1540 (100.0)
1-17	1054 (30.6)	472 (24.8)	582 (37.8)
18-94	2392 (69.4)	1434 (75.2)	958 (62.2)
median (IQR)	*23.0 (14.0-35.0)*	*24.0 (18.0-36.0)*	*23.0 (11.0-35.0)*
Education (n=3,160)*			
No formal education	1847 (58.4)	1262 (70.5)	585 (42.7)
Any schooling	1313 (41.6)	528 (29.5)	785 (57.3)
median (IQR)	*0 (0-2)*	*0 (0-1)*	*0 (0-4)*
Camp location			
Teknaf	455 (13.2)	245 (12.9)	210 (13.6)
Ukhiya	2991 (86.8)	1661 (87.1)	1330 (86.4)
Smoking (n=2,392) †	513 (21.4)	103 (7.2)	410 (42.8)
BCG vaccination	2974 (86.3)	1665 (87.4)	1309 (85.0)
Any relevant symptoms‡	1274 (37.0)	723 (37.9)	551 (35.8)
Any comorbidity§	430 (12.5)	268 (14.1)	162 (10.5)

Note: FDMN, Forcibly Displaced Myanmar Nationals

* Among those aged ≥6 years

† Among those age ≥18 years

‡ Symptoms include fever (20.3%), cough (20.2%), dyspnoea (3.1%), anosmia (1.9%), chills (1.6%), fatigue (10.3%), sore throat (3.9%), headache (10.9%), ageusia (4.5%), nausea (3.9%), vomiting (3.9%), diarrhoea (5.8%), wheezing (1.4%), chest pain (2.9%), rhinorrhoea (11.8%), conjunctivitis (1.1%), other respiratory (0.6%), muscle aches (5.4%), myalgia (3.9%), rash (1.8%), loss appetite (3.7%), nosebleed (0.1%), seizures (0.2%)

§ Comorbidities include cancer (0.1%), diabetes (2.5%), hypertension (5.8%), heart diseases (1.3%), asthma (1.9%), chronic lung disease (0.5%), chronic liver diseases (0.9%), thalassemia or other chronic haematological disorder (0.1%), chronic kidney diseases (0.3%), chronic neurological impairment (0.3%), bone marrow diseases (0.5%)

Seroprevalence of SARS-CoV-2

Overall, 2,090 (928 male and 1,162 female) of 3,446 study participants tested positive for SARS-CoV-2 antibody with the Wantai kit (**Table 2**). The overall crude, weighted, and weighted and test adjusted seroprevalence of SARS-CoV-2 antibodies was 60.7% (95% CI: 59.0–62.3%), 57.4% (95% CI: 55.2–59.7%) and 48.3% (95% CI: 45.3–51.4%), respectively. The distribution of infection (weighted and Kantaro test adjusted) was 57.4 (54.2–60.6) and did not differ between males and females. Children and adolescents (age 1–17 years) had

For peer review only

lower prevalence (38.6%; 95% CI, 33.8–43.4%) compared to the adults (58.1%; 55.2–61.1). There was no significant difference in infection between Teknaf and Ukhiya upazila. Non-smoker had significantly higher prevalence (60.5%; 95% CI: 57.3–63.7) compared to smoker (50.4%; 95% CI: 43.9–56.6%). Almost half (45.7%, 95%CI: 41.9-49.5%) of the asymptomatic participants had positive antibody tests. Participants with at least one comorbidity had higher seroprevalence than those with no comorbidity (57.8%; 95% CI: 50.4–64.5% vs 47.2%; 95% CI: 43.9-50.4%) (**Table 2**). This distribution of seroprevalence across covariates were not different between males and females, except camp location where it was significantly higher among males in Ukhiya, lower among smoker males, higher among symptomatic females and higher among females who reported at least one comorbidity (**Supplemental Table 2**).

For peer review only

Table 2. Crude, weighted and test adjusted over weighted prevalence (95% confidence intervals) of SARS-CoV-2 antibody by sex across covariates, FDMN population, Bangladesh December 2020 (N=3446)

Variable	Count	Unweighted	Weighted†	Weight & test adjusted‡
	Positive/Total	% (95% CI)	% (95% CI)	% (95% CI)
Age, years				
All	2090/3446	60.7 (59.0–62.3)	57.4 (55.2–59.7)	48.3 (45.3–51.4)
1-17	547/1054	51.9 (48.8–55.0)	49.4 (45.6–53.2)	38.6 (33.8–43.4)
18-94	1543/2392	64.5 (62.6–66.4)*	65.5 (63.2–67.8)*	58.1 (55.2–61.1)*
Sex				
Female	1162/1906	61.0 (58.7–63.2)	57.4 (54.2–60.6)	48.3 (44.2–52.3)
Male	928/1540	60.3 (57.8–62.7)	57.4 (54.2–60.6)	48.3 (44.2–52.3)
Education§ (n=3,160)				
No formal education	1147/1847	62.1 (59.8–64.3)	61.7 (58.8–64.5)	53.5 (49.8–57.1)
Any education	842/1313	64.1 (61.5–66.7)	61.8 (58.3–65.2)	53.6 (49.2–57.9)
Camp location				
Teknaf	261/455	57.4 (52.7–62.0)	54.8 (48.9–60.6)	45.1 (37.8–52.2)
Ukhiya	1829/2991	61.2 (59.4–62.9)	58.0 (55.5–60.4)	49.0 (45.8–52.2)
Smoking‖ (n=2,392)				
No	1240/1879	66.0 (63.8–68.1)	67.5 (64.9–70.0)	60.5 (57.3–63.7)
Yes	303/513	59.1 (54.7–63.4)*	59.1 (53.9–64.2)*	50.4 (43.9–56.6)*
BCG vaccination				
No	306/472	64.8 (60.3–69.1)	58.8 (52.7–64.7)	50.0 (42.5–57.3)
Yes	1784/2974	60.0 (58.2–61.8)*	57.2 (54.7–59.6)	48.0 (44.8–51.2)
Any relevant symptoms¶				
No symptom	1282/2172	59.0 (56.9–61.1)	55.3 (52.3–58.2)	45.7 (41.9–49.5)
Any symptom	808/1274	63.4 (60.7–66.1)*	61.2 (57.7–64.6)*	52.9 (48.5–57.2)*
Any comorbidity††				
No comorbidity	1800/3016	59.7 (57.9–61.4)	56.5 (54.0–58.9)	47.2 (43.9–50.4)
Any comorbidity	290/430	67.4 (62.8–71.9)*	65.2 (59.2–70.7)*	57.8 (50.4–64.5)*

Note: FDMN, Forcibly Displaced Myanmar Nationals
* P<0.05 for comparisons between seropositives and categorical factors
† Weighted to FDMN Population size (N=860,697) and distribution published by Refugee Relief and Repatriation Commissioner & United Nations High Commissioner for Refuge, 2020.
‡ Rogan-Gladen formula was used to adjust seroprevalence estimates to account for test sensitivity and specificity of Wantai kits validated by Kantaro [27]
§ Among those age ≥ 6 years
‖ Among those age ≥ 18 years
¶ Symptoms include fever, cough, dyspnoea, anosmia, chills, fatigue, sore throat, headache, ageusia, nausea, vomiting, diarrhoea, wheezing, chest pain, rhinorrhoea, conjunctivitis, other respiratory, muscle aches, myalgia, rash, loss of appetite, nosebleed, seizures
†† Comorbidities include cancer, diabetes, hypertension, heart disease, asthma, chronic lung disease, chronic liver disease, thalassemia or other chronic haematological disorder, chronic kidney disease, chronic neurological impairment, bone marrow disease

The prevalence of SARS- CoV-2 antibody was higher in adults than in children, irrespective of their self-report of symptoms (**Figure 2**). However, prevalence was almost the same in two groups of adults (18-49 years and 50-94 years). Among those having comorbid conditions, the highest seroprevalence was found among participants with self-reported chronic liver disease (67.8%) followed by those with bone marrow disease (66.7%), hypertension (60.6%), diabetes (58.9%), heart disease patients (57.1%) and chronic lung disease/ asthma (52.5%) (**Figure 3**). An increasing seroprevalence was observed with the number of self-reported comorbidities, 55.1% with one comorbidity and 71.8% with three or more comorbidities.

Factors associated with anti-SARS-CoV-2 antibody seropositivity

Univariate logistic regression analysis identified increasing age (OR: 1.02; 95% CI: 1.02–1.03), increasing education level (OR: 1.07; 95% CI: 1.03–1.11), history of relevant symptoms (OR: 1.28; 95% CI: 1.06–1.54) and presence of co-morbid conditions (OR: 1.44; 95% CI: 1.10–1.90) as the factors associated with higher risk of seropositivity, whereas smoking (OR: 0.70; 95% CI: 0.55–0.89) was associated with lower risk of seropositivity. In multiple logistic regression for all subjects adjusted for age, sex, camp locations, BCG vaccination, history of symptoms, and comorbid conditions, the association for higher risk of seropositivity persisted for increasing age (aOR: 1.01; 95% CI: 1.01–1.02) and higher education (aOR: 1.09; 95% CI: 1.04–1.13) only. However, after adding smoking to the model for adults (aged 18–94 years because smoking data were not collected for children), respondents who smoked had a lower risk of infection with SARS-CoV-2 (aOR: 0.63, 95% CI: 0.48–0.83). In addition, respondents who had a history of one or more symptoms had higher odds (aOR: 1.34; 95% CI: 1.08--1.67) of having the infection **(Table 3)**.

For peer review only

Table 3. Risk factors of weighted SARS-CoV-2 infection identified in univariate and multiple weighted logistic regression analysis, FDMN population, Bangladesh December 2020 (N=3446)

Factors	Unadjusted	Adjusted		
		Overall	(Age 1-17 years)	(Age 18-94 years)
	OR (95% CI)	OR (95% CI)	OR (95% CI)	OR (95% CI)
Age (Continuous)	1.02 (1.02–1.03)*	1.01 (1.01–1.02)*	1.09 (1.03–1.15)*	1.01 (0.99–1.01)
Sex				
Female	1	1	1	1
Male	1.00 (0.83–1.20)	1.00 (0.81–1.23)	1.30 (0.89–1.88)	1.05 (0.82–1.34)
Education (continuous)	1.07 (1.03–1.11)*	1.09 (1.04–1.13)*	1.08 (0.97–1.21)	1.06 (1.02–1.11)*
Camp Location				
Teknaf	1	1	1	1
Ukhiya	1.14 (0.88–1.47)	1.10 (0.83–1.46)	1.12 (0.68–1.84)	1.07 (0.78–1.47)
Smoking				
No	1			1
Yes	0.70 (0.55–0.89)*			0.63 (0.48–0.83)*
BCG vaccination				
No	1	1	1	1
Yes	0.94 (0.72–1.22)	0.96 (0.72–1.28)	1.18 (0.55–2.53)	0.85 (0.66–1.12)
Symptoms†				
No	1	1	1	1
Yes	1.28 (1.06–1.54)*	1.20 (0.99–1.47)	1.12 (0.77–1.63)	1.34 (1.08–1.67)*
Comorbidity‡				
No comorbidity	1	1	1	1
One or more	1.44 (1.10–1.90)*	1.10 (0.82–1.48)	1.06 (0.54–2.11)	1.16 (0.85–1.60)

Note: FDMN, Forcibly Displaced Myanmar Nationals
*P<0.05
†Symptoms include fever, cough, dyspnoea, anosmia, chills, fatigue, sore throat, headache, ageusia, nausea, vomiting, diarrhoea, wheezing, chest pain, rhinorrhoea, conjunctivitis, other respiratory, muscle aches, myalgia, rash, loss appetite, nosebleed, seizures
‡Comorbidities include cancer, diabetes, hypertension, heart disease, asthma, chronic lung disease, chronic liver disease, thalassemia or other chronic haematological disorder, chronic kidney disease, chronic neurological impairment, bone marrow disease

For peer review only

Discussions

We report a high seroprevalence of antibodies (total immunoglobulin including both IgG and IgM) to SARS-CoV-2 virus (48.3%) using a cross-sectional survey conducted in December 2020 among FDMNs living in Cox's Bazar district of Bangladesh. This high seroprevalence contrasts with a very low number of infections detected by RT-PCR before the study period. This probably is not surprising as other studies had similar observations even with low seroprevalence, like nationally representative study in Sierra Leone in March 2021 reported weighted seroprevalence of only 2.6%, but it was 43 times higher than the reported number of cases.[29] Seroprevalence study in Pakistan during October to November 2020 although reported a lower seroprevalence of 7.1% based on a low sensitive rapid antibody testing (‘Bioperfectus’ kits for IgG/IgM), the estimated seroprevalence was 62 times higher than the

reported number of cases before the survey period in the sampled districts.[30] High seroprevalence of SARS-CoV-2 IgG (39.2%) was reported from metropolis of the Brazilian Amazon from October 2020 to February 2021, which was also 10 times higher than the case reported by the health authority as of December 2020.[31]

Although we found a higher seroprevalence (58.1%) in adults than children aged 1–17 years (38.6%), there was no difference in seroprevalence for older adults (aged 60 and above years) compared with middle-aged adults (aged 40–59 years), which supports the findings of the systematic review by Levin et al.[32] No difference of seroprevalence between sexes was found through our study. Earlier study in Bangladesh reported that females had a higher rate of both IgG and IgM seropositivity compared with males, but the findings were not significantly different.[13] Similar higher seropositivity among females was reported in Sierra Leone, without statistically significant difference.[29] Seroprevalence study in Pakistan also did not report any significant different results among males and females.[30] Seroprevalence study in South Korea during September 2020 to December 2020 also did not report any differences for sex or age group.[33]

Nearly half 45.7% of the people who did not report any symptom since March 2020 also had evidence of SARS-CoV-2 virus infection. They might have had very mild symptoms to recall correctly or were in fact asymptomatic. The younger age profile of the FDMN population also might explain the higher proportion of mild or asymptomatic infection.[34] Because the current study was conducted during a pandemic, the time of the survey, geographic locations, age and sex composition of the sample, and type of antibody assessment should be considered before judging the findings or comparing it with other studies.

The current study examines SARS-CoV-2 infections among those temporarily or chronically experiencing homelessness (or otherwise living in precarious housing) during the COVID-19 pandemic. The FDMN community has intrinsic differences from the host community, especially regarding awareness of COVID-19 issues and access to related information. Although their access to healthcare was beyond the scope of this survey, understanding the risk and needs of this group is crucial to containing the spread of the pandemic.

Our study findings indicated that by December 2020, almost half of the FDMN people were exposed to the SARS-CoV-2 infections, when population vaccination for COVID-19 yet to be started in the country. Another study in Sitakunda subdistrict in the Chattogram division (Cox's Bazar is also located in this division) of Bangladesh conducted in April 2021 has found 63.1% seroprevalence, which was conducted later than our study so expected to have

For peer review only

more cumulative seropositive population and sample size, geographical context and type of antibody assessment were different than the current study.[35] The capital city of Dhaka also has a very high population density. Another study[13] reported a seroprevalence of IgG of 35.4% in the non-slum population of Dhaka city between April and October 2020. This study, on the other hand, reported 63.5% seroprevalence in slum areas of Dhaka city. Therefore, the high prevalence we found in FDMN is not unusual in the Bangladesh context. A seroprevalence study conducted in eight large slums of Mumbai, India in June–July 2020 reported a seroprevalence of 54.1%. However, the same study reported 16.1% seroprevalence in the non-slum areas of Mumbai.[36] A high seroprevalence of IgG antibodies was also reported in three high density communities in Harare, Zimbabwe during February to April 2021(53.0%)[37] and in urban slums of India during December 2020 to January 2021 (34.7%),[38] both of which might have similar crowded living settings as of FDMN camps.

Like others,[6 39] we have observed a lower seroprevalence in children than adults. During the pandemic, children did not go to schools or any other places for an occupation to acquire the infection. Although most adult women stayed at home (because homemaking was their predominant occupation), they had similar infection rates as their male counterparts. Women might have acquired infections at household levels from their husbands or other male members who might have exposure due to contact with people outside their homes. Imam/priests and incentive volunteers had the highest seroprevalence rates in our series (**Supplemental Figure 2**), similar to other studies.[40] Our results also revealed that non-smokers are likely to have higher seroprevalence than smokers. Contrary to the putative effects of smoking on human health, smoking might have suppressed a due immune response to SARS-CoV-2 infections as reported by few other studies,[41-44] although examining this association was beyond the scope of our study. In addition, conflicting associations of BCG vaccination with SARS-CoV-2 infections have been reported. We did not observe any significant association with BCG vaccination. The proposition of a better cellular response in those who have BCG vaccination[45-47] is beyond the scope of our study.

Our study among FDMN population had several limitations. The studies relying on antibody response have their inherent limitations. The estimates reported here might be affected by the waning antibody levels over time. However, it should be noted that vaccination for SARS-CoV-2 had not been initiated in Bangladesh (and hence among the study population) at the time of the study, and therefore was not likely to influence the seroprevalence estimates. The household roster could be completed for about 71% of targeted subjects (**Supplemental Table 1**) because the listing of households was done several months before the survey. Non-response at the household level was higher than expected (13.6%). This

might have caused overestimation of the seroprevalence, as reflected by the unweighted seroprevalence result in table 2. The population and household level weighting should address the issue of non-response. Again, the Wantai ELISA might have cross-reactivity with antibodies to other common non-SARS-CoV-2 human coronaviruses, which are known to circulate worldwide continuously,[48 49] leading to a false-positive result in some instances. Although we were not able to check for cross-reactivity against other coronaviruses, we adjusted seroprevalence estimates to account for test sensitivity and specificity of Wantai validated by Kantaro to address issue of cross-reactivity from past infection specially during pre-pandemic period from any non-SARS-CoV-2 human corona viruses. Earlier study in Kenya showed slightly lower specificity of Wantai compared with in-house (KWTRP) IgG antibody ELISA kit for pre-pandemic samples, indicating likely overestimation of population seroprevalence.[50] Furthermore, self-reports on comorbid conditions and probable COVID-19 symptoms due to a long recall period might impact our study results. Additionally, as the study protocol has been adopted from WHO unity study, we did not include questions on non-pharmaceutical public health measures, e.g. use of masks which can influence the population seroprevalence as reported by other studies in Bangladesh.[51]

Conclusions

The findings of this study imply that the prevalence of seropositivity is likely to be much higher than the presumed RT-PCR confirmed rates in FDMN population. A high seroprevalence across age and sex groups, irrespective of a history of symptoms, may indicate an inadequacy of infection control measures. Because of a very high population density in the camps, social distancing may not work. Other well-known public health measures, such as face masks and mass vaccination, are warranted.

Data Availability Statement

To protect patients' privacy, the ethical approval obtained for this study prevents human data from being disclosed publicly. Requests to access the datasets should be directed to Professor Tahmina Shirin, Director, IEDCR, email: director@iedcr.gov.bd; directoriedcr@gmail.com.

Ethics statements

Patient consent for publication

Not applicable

For peer review only

Ethics approval

The study protocol was submitted and approved by the Institutional Review Board of the Institute of Epidemiology, Disease Control and Research (Approval Memo No. IEDCR/IRB/2020/24). The participants provided their written informed consent and /or assent (as applicable) to participate in this study. A thumbprint was given if someone was unable to sign.

Acknowledgements

We gratefully acknowledge the field team, MOHFW, DGHS, divisional coordinators, civil surgeons, Upazila health and family planning officers and health assistants for their support. We are also thankful to the following local and international NGO and development partners: WHO, RRRC, UNHCR; Relief International; FH/MTI; ICRC/BDRCS; BRAC; icddr,b; World Bank; Bureau of Population, Refugees, and Migration – United States Department of State through Driving Public Health Action in the World's Largest Refugee Camps; Government of Norway. We are indebted to the camp community leaders (M*ajhis*) to facilitate the data collection and FDMN camp residents for their participation with the study.

Authors Contributions

MR, EE, MMZ, TS and MSF conceptualised this study. MR, ASMA, DSK, FH, EE, ANA, MMZ, TS and MSF developed the methodology and study design. MR, SRK, DSK, FH, EE, NA and MMZ wrote the protocol. MR, SRK, DSK, FH, EE, NA, MMB and MKA developed the data collection tool. DSK and RB developed the software used. ANA, MMZ, TS and MSF validated analyses, data and methodology used. MR, DSK, MSI and RB conducted the formal analysis. MR, SRK, ASMA, DSK, FH, ANA, NA, SS, DP, RB, RI, AM, MS, MMB, ARS, MHK, MMZ and MSF undertook the investigation. ASMA, ANA, KVH, MMZ and TS provided resources for the study. MR, DSK and MSI curated the data. MR, DSK, FH, MSI and MMZ wrote the original draft of the manuscript. MSI designed the visualisations used. MR, SRK, ASMA, FH, ANA, NA, DP, RI, AM, MMB, KVH, MMZ, TS and MSF provided supervision. MR, SRK, ASMA, DSK, FH, NA, DP, RI, AM, MS, ARS, MKA, KVH, MMZ, TS and MSF administered this project. MMZ, TS and MSF acquired the funding for the study. All authors participated in reviewing and editing the manuscript.

Funding

This work was supported by the World Health Organization (Ref. No. 2020/1060317-2). However, no fund has been used to prepare this manuscript.

Competing interests

None declared.

The authors alone are responsible for their views expressed in this article, which do not necessarily represent the institutions' views, decisions, or policies with which they are affiliated.

References

1. World Health Organization. Novel Coronavirus (2019-nCoV): situation report, 11. Geneva: World Health Organization, 2020.
2. World Health Organization. Coronavirus disease (COVID-19): Serology, antibodies and immunity: Accessed; 2021 [Available from: https://www.who.int/news-room/questions-and-answers/item/coronavirus-disease-covid-19-serology.
3. Verity R, Okell LC, Dorigatti I, et al. Estimates of the severity of coronavirus disease 2019: a model-based analysis. *The Lancet infectious diseases* 2020;20(6):669-77.
4. World Health Organization. Population-based age-stratified seroepidemiological investigation protocol for COVID-19 virus infection, 17 March 2020: World Health Organization, 2020.
5. Bendavid E, Mulaney B, Sood N, et al. Covid-19 antibody seroprevalence in santa clara county, california. *International journal of epidemiology* 2021;50(2):410-19.
6. Havers FP, Reed C, Lim T, et al. Seroprevalence of antibodies to SARS-CoV-2 in 10 sites in the United States, March 23-May 12, 2020. *JAMA internal medicine* 2020;180(12):1576-86.
7. Montenegro P, Brotons C, Serrano J, et al. Community seroprevalence of COVID-19 in probable and possible cases at primary health care centres in Spain. *Family Practice* 2021;38(2):153-58.
8. Anna F, Goyard S, Lalanne AI, et al. High seroprevalence but short-lived immune response to SARS-CoV-2 infection in Paris. *European journal of immunology* 2021;51(1):180-90.
9. Nisar MI, Ansari N, Khalid F, et al. Serial population-based serosurveys for COVID-19 in two neighbourhoods of Karachi, Pakistan. *International Journal of Infectious Diseases* 2021;106:176-82.
10. Inbaraj LR, George CE, Chandrasingh S. Seroprevalence of COVID-19 infection in a rural district of South India: A population-based seroepidemiological study. *PloS one* 2021;16(3):e0249247.
11. Chimeddorj B, Mandakh U, Le L-V, et al. SARS-CoV-2 seroprevalence in Mongolia: Results from a national population survey. *The Lancet Regional Health-Western Pacific* 2021;17:100317.
12. Jubayer F, Kayshar S, Limon TI. First COVID-19 case in the Rohingya camp in Bangladesh: Needs proper attention. *Public Health* 2021;191:20.
13. Bhuiyan TR, Akhtar M, Akter A, et al. Seroprevalence of SARS-CoV-2 antibodies in Bangladesh related to novel coronavirus infection. *IJID Regions* 2022;2:198-203.
14. Shirin T, Bhuiyan TR, Charles RC, et al. Antibody responses after COVID-19 infection in patients who are mildly symptomatic or asymptomatic in Bangladesh. *International Journal of Infectious Diseases* 2020;101:220-25.
15. Uddin Md Zahed I, Jenkins B. The Politics of Rohingya Ethnicity: Understanding the Debates on Rohingya in Myanmar. *Journal of Muslim Minority Affairs* 2022;42(1):117-35. doi: 10.1080/13602004.2022.2064054
16. Islam MM, Khan MN, Rahman MM. Factors affecting child marriage and contraceptive use among Rohingya girls in refugee camps. *The Lancet Regional Health-Western Pacific* 2021;12:100175.
17. World Health Organization Bangladesh. Emergency: Rohingya Crisis-Monthly Situation Report # 3. https://www.who.int/bangladesh/emergencies/Rohingyacrisis/situation-report---rohingya-crisis, 2022.
18. Truelove S, Abrahim O, Altare C, et al. The potential impact of COVID-19 in refugee camps in Bangladesh and beyond: A modeling study. *PLoS medicine* 2020;17(6):e1003144.
19. World Health Organization Bangladesh. Emergency: Rohingya Crisis- Bi-weekly Situation Report #25. https://www.who.int/docs/default-source/searo/bangladesh/bangladesh---rohingya-crisis---pdf-reports/sitreps/2019/bi-weekly-situation-report-25---19-december-2019.pdf?sfvrsn=4241b323_2, 2019.

For peer review only

20. Inter Sector Coordination Group. Joint Multi-Sector Needs Assessment (J-MSNA), Rohingya Refugees, Cox's Bazar, Bangladesh 2020 [Available from: https://www.humanitarianresponse.info/sites/www.humanitarianresponse.info/files/documents/files/2021_05_iscg_2020_msna_report_refugee_english.pdf.
21. United Nations High Commissioner for Refugees (UNHCR). Joint Government of Bangladesh - UNHCR Population factsheet as of 31 August 2020. Population factsheet. https://data.unhcr.org/en/documents/details/78973: UNHCR.
22. OpenEpi: Open Source Epidemiologic Statistics for Public Health. Version 2.3.1; 2013.
23. Initiative HH. KoBoToolbox. *Retrieved November 14, 2017, from http://www kobotoolbox org* 2021
24. Lassaunière R, Frische A, Harboe ZB, et al. Evaluation of nine commercial SARS-CoV-2 immunoassays. *MedRxiv* 2020
25. KANTARO. Kantaro's SARS-CoV-2 IgG Antibody Test Kits https://kantarobio.com/antibody-test-kits2021 [2022.
26. Hakim F, Bhuiyan R, Akter MK, et al. Weighting National Survey Data in Bangladesh: Why, How and Which weight? Weighting survey data. *Bangladesh Medical Research Council Bulletin* 2022;47(2):118-26. doi: 10.3329/bmrcb.v47i2.57769
27. Rogan WJ, Gladen B. Estimating prevalence from the results of a screening test. *American journal of epidemiology* 1978;107(1):71-76.
28. Henrion MY. bootComb—an R package to derive confidence intervals for combinations of independent parameter estimates: Oxford University Press, 2021.
29. Barrie MB, Lakoh S, Kelly JD, et al. SARS-CoV-2 antibody prevalence in Sierra Leone, March 2021: a cross-sectional, nationally representative, age-stratified serosurvey. *BMJ Glob Health* 2021;6(11):e007271. doi: 10.1136/bmjgh-2021-007271
30. Ahmad AM, Shahzad K, Masood M, et al. COVID-19 seroprevalence in Pakistan: a cross-sectional study. *BMJ open* 2022;12(4):e055381. doi: 10.1136/bmjopen-2021-055381 [published Online First: 2022/04/08]
31. da Silva Torres MK, Lopes FT, de Lima ACR, et al. Seroprevalence and risk factors for COVID-19 in the metropolis of the Brazilian Amazon. *Scientific reports* 2022;12(1):8571. doi: 10.1038/s41598-022-12629-z [published Online First: 2022/05/21]
32. Levin AT, Owusu-Boaitey N, Pugh S, et al. Assessing the burden of COVID-19 in developing countries: systematic review, meta-analysis and public policy implications. *BMJ Glob Health* 2022;7(5):e008477. doi: 10.1136/bmjgh-2022-008477
33. Nah E-H, Cho S, Park H, et al. Nationwide seroprevalence of antibodies to SARS-CoV-2 in asymptomatic population in South Korea: a cross-sectional study. *BMJ open* 2021;11(4):e049837. doi: 10.1136/bmjopen-2021-049837
34. Poletti P, Tirani M, Cereda D, et al. Association of age with likelihood of developing symptoms and critical disease among close contacts exposed to patients with confirmed SARS-CoV-2 infection in Italy. *JAMA network open* 2021;4(3):e211085-e85.
35. Bhuiyan TR, Hulse JD, Hegde ST, et al. SARS-CoV-2 seroprevalence before Delta variant surge, Chattogram, Bangladesh, March–June 2021. *Emerging infectious diseases* 2022;28(2):429.
36. Malani A, Shah D, Kang G, et al. Seroprevalence of SARS-CoV-2 in slums versus non-slums in Mumbai, India. *The Lancet Global Health* 2021;9(2):e110-e11.
37. Fryatt A, Simms V, Bandason T, et al. Community SARS-CoV-2 seroprevalence before and after the second wave of SARS-CoV-2 infection in Harare, Zimbabwe. *EClinicalMedicine* 2021;41 doi: 10.1016/j.eclinm.2021.101172
38. Murhekar MV, Bhatnagar T, Thangaraj JWV, et al. SARS-CoV-2 seroprevalence among the general population and healthcare workers in India, December 2020–January 2021. *International Journal of Infectious Diseases* 2021;108:145-55. doi: https://doi.org/10.1016/j.ijid.2021.05.040

For peer review only

39. Stringhini S, Wisniak A, Piumatti G, et al. Seroprevalence of anti-SARS-CoV-2 IgG antibodies in Geneva, Switzerland (SEROCoV-POP): a population-based study. *The Lancet* 2020;396(10247):313-19.
40. Lopez-Pena P, Davis CA, Mobarak AM, et al. Prevalence of COVID-19 symptoms, risk factors, and health behaviors in host and refugee communities in Cox's Bazar: A representative panel study. *Bulletin of the World Health Organization* 2020;11
41. Alali WQ, Bastaki H, Longenecker JC, et al. Seroprevalence of SARS-CoV-2 in migrant workers in Kuwait. *Journal of travel medicine* 2021;28(2):taaa223.
42. Barchuk A, Skougarevskiy D, Titaev K, et al. Seroprevalence of SARS-CoV-2 antibodies in Saint Petersburg, Russia: a population-based study. *Scientific reports* 2021;11(1):1-9.
43. Carrat F, de Lamballerie X, Rahib D, et al. Seroprevalence of SARS-CoV-2 among adults in three regions of France following the lockdown and associated risk factors: a multicohort study. 2020
44. Roederer T, Mollo B, Vincent C, et al. Seroprevalence and risk factors of exposure to COVID-19 in homeless people in Paris, France: a cross-sectional study. *The Lancet Public Health* 2021;6(4):e202-e09.
45. Rivas MN, Ebinger JE, Wu M, et al. BCG vaccination history associates with decreased SARS-CoV-2 seroprevalence across a diverse cohort of health care workers. *The Journal of clinical investigation* 2021;131(2)
46. Escobar LE, Molina-Cruz A, Barillas-Mury C. BCG vaccine protection from severe coronavirus disease 2019 (COVID-19). *Proceedings of the National Academy of Sciences* 2020;117(30):17720-26.
47. Garzon-Chavez D, Rivas-Condo J, Echeverria A, et al. COVID-19 infection and previous bcg vaccination coverage in the Ecuadorian population. *Vaccines* 2021;9(2):91.
48. Ogimi C, Kim YJ, Martin ET, et al. What's New With the Old Coronaviruses? *J Pediatric Infect Dis Soc* 2020;9(2):210-17. doi: 10.1093/jpids/piaa037 [published Online First: 2020/04/22]
49. Grimwood K, Lambert SB, Ware RS. Endemic Non–SARS-CoV-2 Human Coronaviruses in a Community-Based Australian Birth Cohort. *Pediatrics* 2020;146(5) doi: 10.1542/peds.2020-009316
50. Nyagwange J, Kutima B, Mwai K, et al. Comparative performance of WANTAI ELISA for total immunoglobulin to receptor binding protein and an ELISA for IgG to spike protein in detecting SARS-CoV-2 antibodies in Kenyan populations. *Journal of Clinical Virology* 2022;146:105061. doi: https://doi.org/10.1016/j.jcv.2021.105061
51. Raqib R, Sarker P, Akhtar E, et al. Seroprevalence of SARS-CoV-2 infection and associated factors among Bangladeshi slum and non-slum dwellers in pre-COVID-19 vaccination era: October 2020 to February 2021. *PloS one* 2022;17(5):e0268093. doi: 10.1371/journal.pone.0268093

For peer review only

Figure Legends:

Figure 1. Flow diagram showing selection of respondents of the FDMN population, Bangladesh December 2020

Note: FDMN, Forcibly Displaced Myanmar Nationals

*Estimated from FDMN population frame of Refugee Relief and Repatriation Commissioner (RRRC) & United Nations High Commissioner for Refuge (UNHCR)

Figure 2. Age-specific weighted test adjusted seroprevalence in male and female participants with or without history of symptoms, FDMN population, Bangladesh December 2020 (N=3446): Panel A: female, Panel B: male & Panel C: all subjects. Error bars indicate 95% confidence intervals

Note: FDMN, Forcibly Displaced Myanmar Nationals. Weighted to FDMN Population size (N=860,697) and distribution published by Refugee Relief and Repatriation Commissioner & United Nations High Commissioner for Refuge, 2020. Rogan-Gladen formula was used to adjust seroprevalence estimates to account for test sensitivity and specificity of Wantai kits validated by Kantaro[25]

Figure 3. Weighted test adjusted seroprevalence of SARS-CoV-2 among self-reported comorbid conditions, FDMN population, Bangladesh December 2020 (N=3446): Panel A: type of comorbidity, Panel B: number of comorbidities. Error bars indicate 95% confidence intervals

Note: FDMN, Forcibly Displaced Myanmar Nationals. Weighted to FDMN Population size (N=860,697) and distribution published by Refugee Relief and Repatriation Commissioner & United Nations High Commissioner for Refuge, 2020. Rogan-Gladen formula was used to adjust seroprevalence estimates to account for test sensitivity and specificity of Wantai kits validated by Kantaro[25]

For peer review only

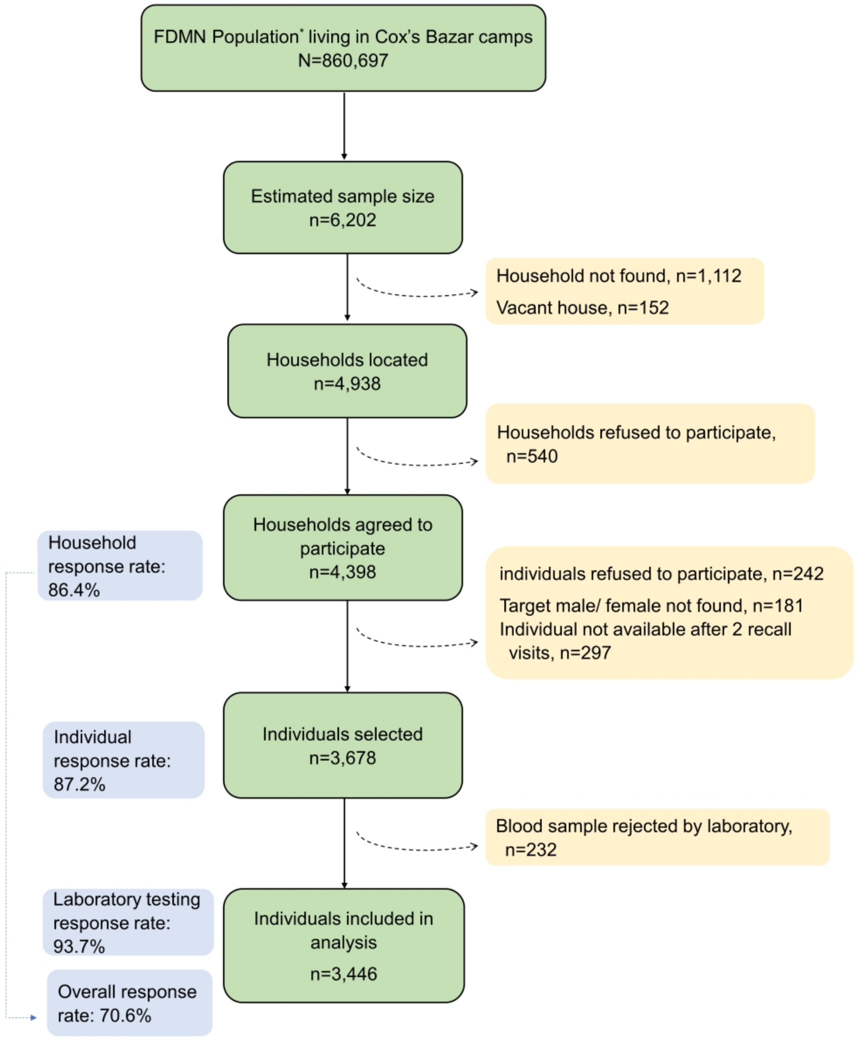

Figure 1. Flow diagram showing selection of respondents of the FDMN population, Bangladesh December 2020

Note: FDMN, Forcibly Displaced Myanmar Nationals
*Estimated from FDMN population frame of Refugee Relief and Repatriation Commissioner (RRRC) & United Nations High Commissioner for Refuge (UNHCR)

338x414mm (300 x 300 DPI)

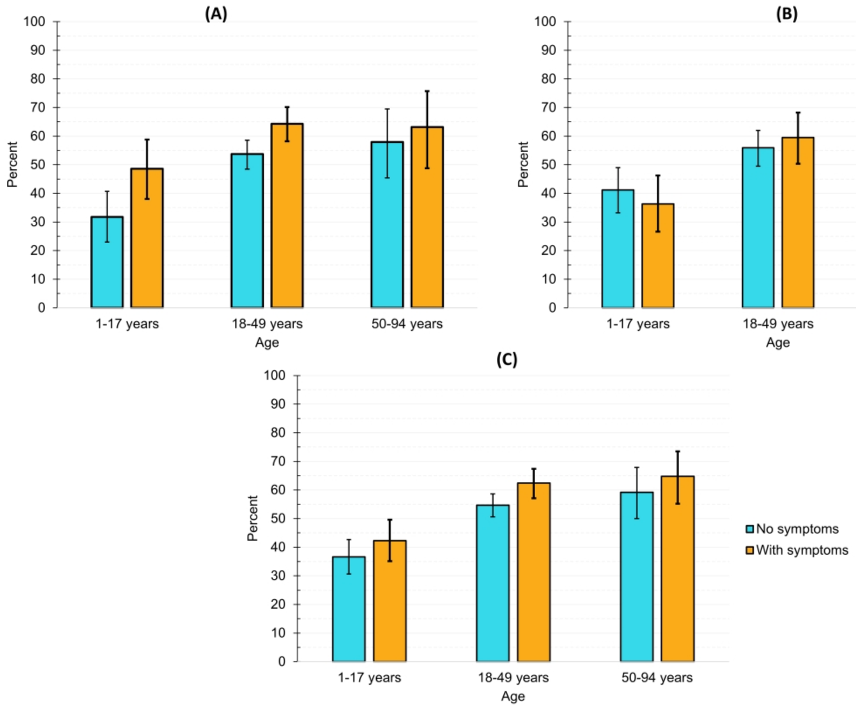

Figure 2. Age-specific weighted test adjusted seroprevalence in male and female participants with or without history of symptoms, FDMN population, Bangladesh December 2020 (N=3446): Panel A: female, Panel B: male & Panel C: all subjects. Error bars indicate 95% confidence intervals

Note: FDMN, Forcibly Displaced Myanmar Nationals. Weighted to FDMN Population size (N=860,697) and distribution published by Refugee Relief and Repatriation Commissioner & United Nations High Commissioner for Refuge, 2020. Rogan-Gladen formula was used to adjust seroprevalence estimates to account for test sensitivity and specificity of Wantai kits validated by Kantaro[25]

338x240mm (300 x 300 DPI)

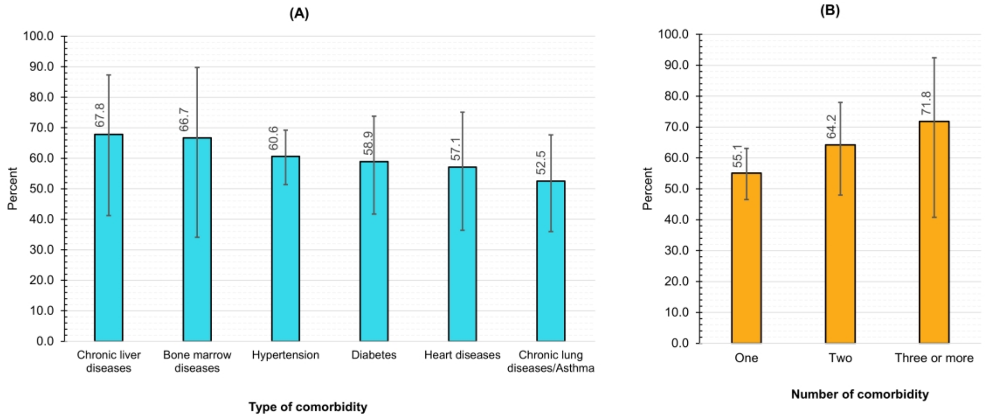

Figure 3. Weighted test adjusted seroprevalence of SARS-CoV-2 among self-reported comorbid conditions, FDMN population, Bangladesh December 2020 (N=3446): Panel A: type of comorbidity, Panel B: number of comorbidities. Error bars indicate 95% confidence intervals

Note: FDMN, Forcibly Displaced Myanmar Nationals. Weighted to FDMN Population size (N=860,697) and distribution published by Refugee Relief and Repatriation Commissioner & United Nations High Commissioner for Refuge, 2020. Rogan-Gladen formula was used to adjust seroprevalence estimates to account for test sensitivity and specificity of Wantai kits validated by Kantaro[25]

338x150mm (300 x 300 DPI)

Supplemental Material

Seroprevalence of SARS-CoV-2 antibodies among Forcibly Displaced Myanmar Nationals in Cox's Bazar, Bangladesh 2020: a population-based cross-sectional study

Mahbubur Rahman[1*], Samsad Rabbani Khan [1], A S M Alamgir [1], David S. Kennedy [2], Ferdous Hakim [3], Egmond Samir Evers[2], Nawroz Afreen [1], Ahmed Nawsher Alam [1], Md Sahidul Islam [3], Debashish Paul [2], Rijwan Bhuiyan [4], Raisul Islam [2], Adneen Moureen [5], M Salimuzzaman [1], Mallick Masum Billah [1], Ahmed Raihan Sharif [1], Mst. Khaleda Akter [3], Sharmin Sultana [1], Manjur Hossain Khan [1], Kai Von Harbou [2], Mohammad Mostafa Zaman [3], Tahmina Shirin [1], Meerjady Sabrina Flora [6]

[1]Institute of Epidemiology, Disease Control and Research (IEDCR), Dhaka, Bangladesh

[2]WHO Emergency Sub-Office, World Health Organization, Cox's Bazar, Bangladesh

[3]Research and Publication, World Health Organization, Dhaka, Bangladesh

[4]Coordination Center, Ministry of Health and Family Welfare, Cox's Bazar, Bangladesh

[5]IEDCR Field Laboratory, World Health Organization, Cox's Bazar, Bangladesh

[6]Directorate General of Health Services (DGHS), Dhaka, Bangladesh

Contents

Weighted and test adjusted prevalence 2

Bootstrapping for confidence interval 3

Supplementary Table 1. Responses at household, individual and laboratory testing levels, FDMN seroprevalence survey, Bangladesh December 2020 4

Supplementary Table 2: test adjusted over weighted SARS-CoV-2 antibody prevalence by sex across covariates, FDMN population, Bangladesh 2020 5

Supplementary Figure 1. FDMN camps in Ukhiya and Teknaf of Cox's Bazar district, Bangladesh December 2020 6

Supplementary Figure 2: Test adjusted over weighted seroprevalence among respondent occupation, FDMN population, Bangladesh December 2020 7

For peer review only

Weighted and test adjusted prevalence

Primarily we used WANTAI total antibody SARS-CoV-2 ELISA kits to estimate the seroprevalence, which has some limitations and could result in false positives and false negatives estimates. In order to validate the test result, we used Kantaro IgG ELISA test kit. We took the following approach to adjust the seroprevalence of WANTAI by KANTARO kits.

Let $\pi = P(K^+)$ represent the population prevalence of antibodies to SARS-CoV-2 and let $p = P(W^+)$ be the proportion of participants who test positive in WANTAI. Let, $s_e = P(W^+|K^+)$ be the sensitivity of the test (the probability of testing positive in WANTAI given an individual is positive in KANTARO), and let, $s_p = P(K^-)$ be the specificity of the test (the probability of testing negative in WANTAI given an individual is negative in KANTARO). We can write the expected fraction of positive tests, *p*, as follows:

$$p = \pi s_e + (1-\pi)(1-s_p)$$

Therefore, the test adjusted estimated seroprevalence is expressed using following formula [1]-

$$\pi = \frac{p-(1-s_p)}{s_e\,(1-s_p)} \quad \text{……………………………………………….} \quad (1)$$

Of 3,446 total samples in our study, we found 2,090 positives for WANTAI. Again, we used KANTARO quantitative test to those WANTAI positive samples and we found 290 negatives. Since we didn't test any negative WANTAI samples, and we assumed that all negative samples in WANTAI are also negative in KANTARO. A confusion matrix is displayed below-

	Kantaro			
Wantai		p	n	Total
	p	1800	290	2090
	n	0	1356	1356
	Total	1800	1646	3046

Table: Confusion Matrix between WANTAI and KANTARO where *'p'* denoting the positive and *'n'* denoting the negative test result.

Sensitivity, $s_e = \frac{1800}{1800+0} = 1.00$, and specificity, $s_p = \frac{1356}{290+1356} = 0.8238$

Therefore, overall seroprevalence, $\pi = \frac{0.574-(1-0.8238)}{1-(1-0.8238)} = 0.4829$

For peer review only - http://bmjopen.bmj.com/site/about/guidelines.xhtml

Bootstrapping for confidence interval

We calculated confidence intervals on our weighted sample prevalence estimates based on a non-parametric highest density interval (HDI) bootstrap [2]. Our bootstrap procedure resamples data from our actual datasets for sensitivity, specificity, and prevalence. We use the following steps-

- First, we draw a single bootstrap sample (i.e. with replacement) from the beta distribution of the sensitivity data. For this sample, we calculate the mean value of sensitivity. Let s_{e_j} represent the value for the *jth* iteration of this procedure.
- First, we draw a single bootstrap sample (i.e. with replacement) from the beta distribution of the specificity data. For this sample, we calculate the mean value of specificity. Let s_{p_j} represent the value for the *jth* iteration of this procedure.
- For our weighted estimate, we calculate weighted prevalence of WANTAI in the bootstrap sample . Let p_j represent this value for the jth iteration of this procedure.
- We then calculate the test adjusted prevalence of antibodies to SARS-CoV-2 for the *jth* bootstrap sample using the equation (1)
- Repeat the steps above for j=1...1,000,000 bootstrap samples.
- In our tables, we report the highest density interval (HDI) of the distributions of π_j over the 1,000,000 bootstrap samples as the lower and upper ends of the 95% bootstrap confidence intervals.

The confidence intervals for the test-adjusted estimates were derived using bootstrap sampling, with 1,000,000 parametric bootstrap samples for each estimate, using the "*adjPrevSensSpecCI*" function of the "*bootComb*" R package.

References

1. Chimeddorj B, Mandakh U, Le LV, Byambasogt B, Deleg Z, Enebish O, Altanbayar O, Magvan B, Gantumur A, Byambaa O, Enebish G. SARS-CoV-2 seroprevalence in Mongolia: Results from a national population survey. The Lancet Regional Health-Western Pacific. 2021 Dec 1;17:100317.
2. Bendavid E, Mulaney B, Sood N, Shah S, Bromley-Dulfano R, Lai C, Weissberg Z, Saavedra-Walker R, Tedrow J, Bogan A, Kupiec T. Covid-19 antibody seroprevalence in santa clara county, california. International journal of epidemiology. 2021 Apr;50(2):410-9.
3. Henrion MY. bootComb—an R package to derive confidence intervals for combinations of independent parameter estimates.

Supplemental Table 1. Responses at household, individual and laboratory testing levels, FDMN seroprevalence survey, Bangladesh December 2020

Responses	All	
	**n**	**%**
At household level		
Roster completed (RC)	4398	70.9
Vacant house/ No HH respondent available (VH)	152	2.5
House not found*	1112	17.9
Refused interview (RefI)	540	8.7
Total	6202	100
Household response rate (HRR)†		86.4
At individual level		
Completed (C)	3678	83.6
No one eligible*	181	4.1
Unavailable (U)	297	6.8
Refused (R)	242	5.5
Total	4398	100
Individual response rate (IRR)‡		87.2
At laboratory testing level		
Laboratory testing completed (C)	3446	93.7
Sample rejected by laboratory (Rej)	232	6.3
Total	3678	100
Laboratory testing response rate (LTRR)§		93.7
Overall response rate¶		70.6

Note: FDMN, Forcibly Displaced Myanmar Nationals
*Not included as sample as they do not qualify[1] as sample for the Survey
†Household response rate (%)=[RCx100]/[RC+VH+RefI]

‡Individual response rate (%)=[Cx100]/[C+II+U+R+RI]

§Laboratory testing response rate (%)=[Cx100]/[C+Rej]

¶Overall response rate (%)=HRR*IRR*LTRR/(100*100)

[1]The American Association for Public Opinion Research. 2016. Standard Definitions: Final Dispositions of Case Codes and Outcome Rates for Surveys. 9th edition. AAPOR. Available from: https://www.aapor.org/AAPOR_Main/media/publications/Standard-Definitions20169theditionfinal.pdf (Accessed on 3 September 2019)

For peer review only

Supplemental Table 2: test adjusted over weighted SARS-CoV-2 antibody prevalence by sex across covariates, FDMN population, Bangladesh 2020

Variable	Weighted prevalence, % (95% CI)			Weighted & kit adjusted prevalence, % (95% CI)		
	Overall	Female	Male	Overall	Female	Male
Age, years						
All	57.4 (55.1-59.7)	57.4 (54.2-60.6)	57.4 (54.2-60.6)	48.3 (45.3-51.4)	48.3 (44.2-52.3)	48.3 (44.2-52.3)
1-17	49.4 (45.6-53.2)	48.6 (43.0-54.3)	50.2 (45.1-55.2)	38.6 (33.8-43.4)	37.6 (30.7-44.7)	39.5 (33.2-45.8)
18-94	65.5 (63.2-67.8)*	65.4 (62.4-68.2)*	65.7 (62.0-69.3)*	58.1 (55.2-61.1)*	58.0 (54.3-61.6)*	58.4 (53.8-62.9)*
18-49	64.9 (62.3-67.4)	65.1 (61.9-68.1)	64.7 (60.4-68.8)	57.4 (54.1-60.6)	57.6 (53.6-61.4)	57.1 (51.9-62.3)
50-94	68.5 (63.0-73.5)*	67.2 (59.5-74.2)*	69.5 (61.7-76.3)*	61.8 (55.1-68.0)*	60.2 (50.9-68.9)*	63.0 (53.7-71.4)*
Education† (n=3,160)						
No formal education	61.7 (58.8-64.5)	61.4 (57.9-64.8)	62.1 (57.0-67.0)	53.5 (49.8-57.1)	53.1 (48.8-57.4)	54.0 (47.7-60.1)
Primary above	61.8 (58.3-65.2)	59.0 (53.0-64.8)	63.8 (59.5-67.9)	53.6 (49.2-57.9)	50.2 (42.9-57.4)	56.1 (50.8-61.1)
Camp location						
Teknaf	54.8 (48.9-60.6)	59.7 (51.0-67.7)	49.4 (41.3-57.6)	45.1 (37.8-52.2)	51.1 (40.6-60.9)	38.6 (28.7-48.7)
Ukhiya	58.0 (55.5-60.4)	56.9 (53.5-60.3)	59.1 (55.5-62.5)*	49.0 (45.8-52.2)	47.7 (43.3-51.9)	50.4 (45.8-54.6)*
Smoking‡ (n=2,392)						
No	67.5 (64.9-70.0)	65.3 (62.2-68.2)	71.8 (67.1-76.1)	60.5 (57.3-63.7)	57.9 (54.0-61.5)	65.8 (60.1-71.2)
Yes	59.1 (53.9-64.2)*	66.5 (55.6-75.9)	57.5 (51.6-63.2)*	50.4 (43.9-56.6)*	59.3 (46.5-71.2)	48.4 (41.2-55.5)*
BCG vaccination						
No	58.8 (52.7-64.7)	58.9 (50.0-67.2)	58.7 (50.2-66.7)	50.0 (42.5-57.3)	50.1 (39.4-60.4)	49.9 (39.5-59.7)
Yes	57.2 (54.7-59.6)	57.2 (53.8-60.6)	57.2 (53.7-60.6)	48.0 (44.8-51.2)	48.0 (43.8-52.4)	48.0 (43.6-52.3)
Any relevant symptoms						
No symptom	55.3 (52.3-58.2)	53.3 (49.1-57.4)	57.3 (53.2-61.4)	45.7 (41.9-49.5)	43.3 (38.1-48.5)	48.2 (43.1-53.3)
Any symptom	61.2 (57.7-64.6)*	64.6 (59.9-69.1)*	57.5 (52.3-62.5)	52.9 (48.5-57.2)*	57.0 (51.2-62.6)*	48.4 (42.0-54.6)
Any comorbidity						
No comorbidity	56.5 (54.0-58.9)	56.1 (52.6-59.5)	56.9 (53.5-60.2)	47.2 (43.9-50.4)	46.7 (42.3-51.0)	47.7 (43.4-51.9)
Any comorbidity	65.2 (59.2-70.7)*	67.3 (59.8-74.0)*	62.4 (52.4-71.4)	57.8 (50.4-64.5)*	60.3 (51.4-68.7)*	54.4 (42.4-65.5)

Note: FDMN, Forcibly Displaced Myanmar Nationals
*P<0.05
†Among those aged 6 years or more
‡Among those aged 18 years or more

For peer review only

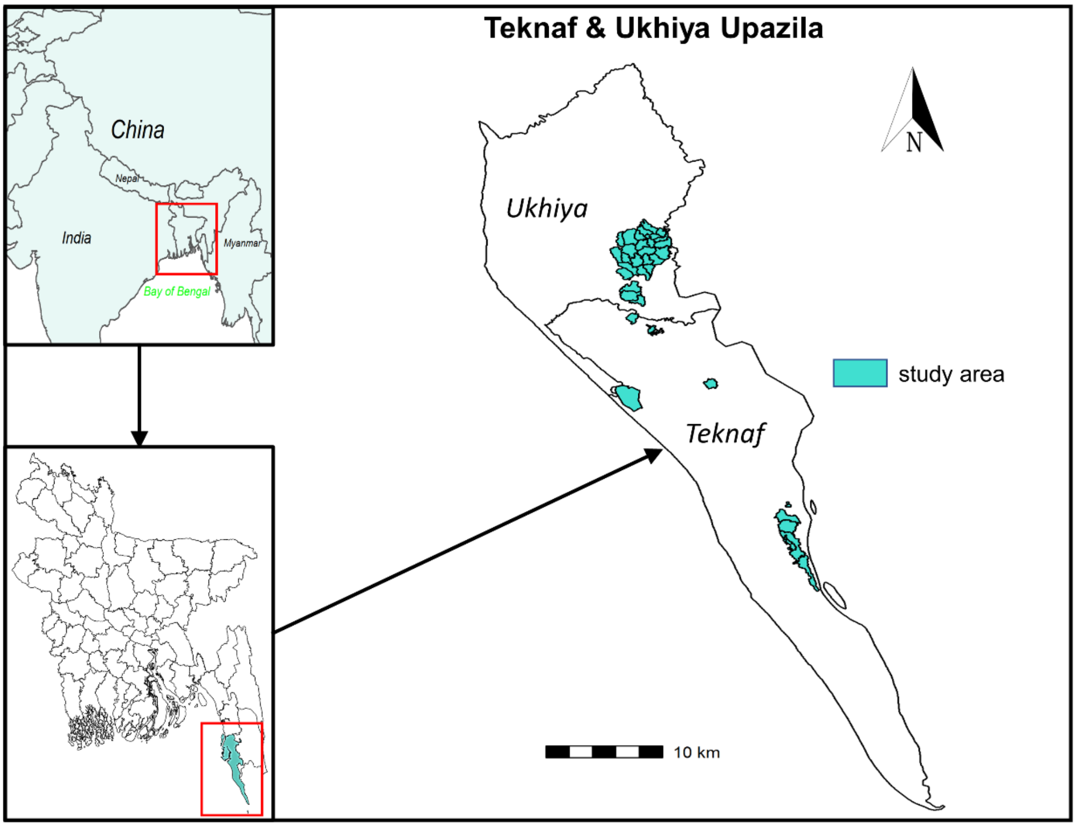

Supplemental Figure 1. FDMN camps in Ukhiya and Teknaf of Cox's Bazar district, Bangladesh December 2020

Note: FDMN, Forcibly Displaced Myanmar Nationals

review only

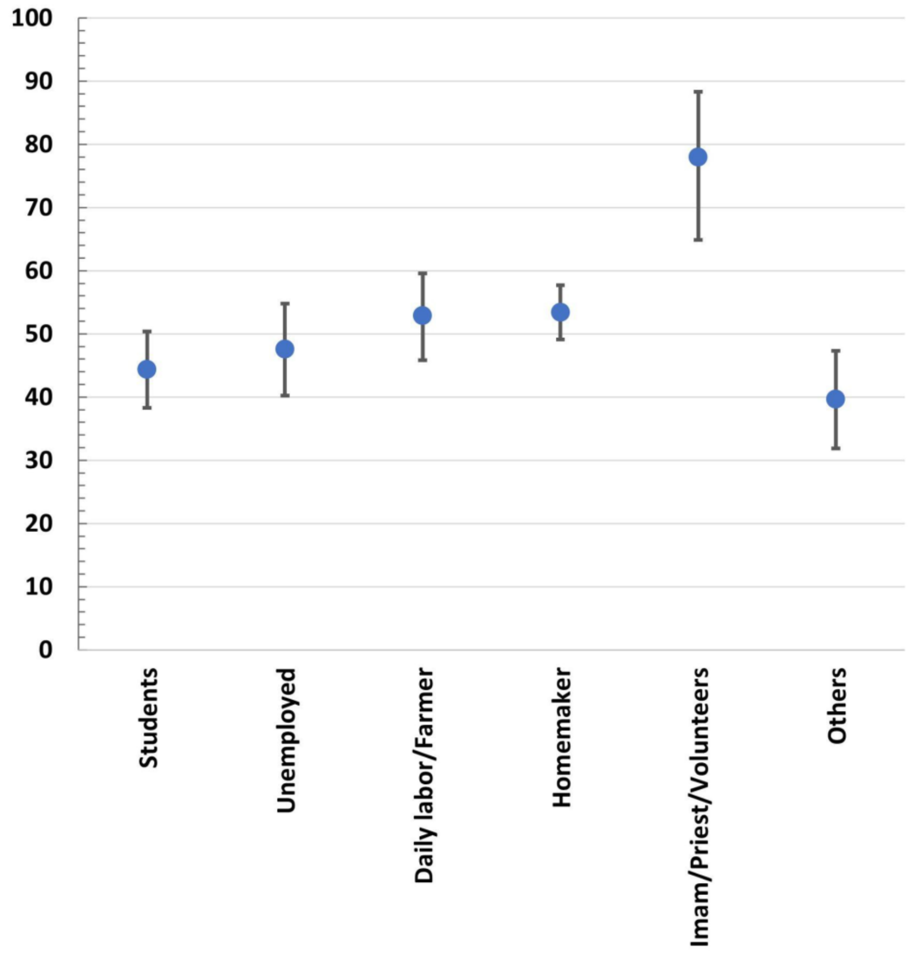

Supplemental Figure 2: Test adjusted over weighted seroprevalence among respondent occupation, FDMN population, Bangladesh December 2020

Note: Blue dot represents the prevalence and error bar indicating confidence interval.

STROBE Statement—Checklist of items that should be included in reports of ***cross-sectional studies***

	Item No	Recommendation	Page No
**Title and abstract**	1	(*a*) Indicate the study's design with a commonly used term in the title or the abstract	3, 4
		(*b*) Provide in the abstract an informative and balanced summary of what was done and what was found	4-5
**Introduction**			
Background/rationale	2	Explain the scientific background and rationale for the investigation being reported	7
Objectives	3	State specific objectives, including any prespecified hypotheses	7
**Methods**			
Study design	4	Present key elements of study design early in the paper	8
Setting	5	Describe the setting, locations, and relevant dates, including periods of recruitment, exposure, follow-up, and data collection	8
Participants	6	(*a*) Give the eligibility criteria, and the sources and methods of selection of participants	8
Variables	7	Clearly define all outcomes, exposures, predictors, potential confounders, and effect modifiers. Give diagnostic criteria, if applicable	8-9
Data sources/ measurement	8*	For each variable of interest, give sources of data and details of methods of assessment (measurement). Describe comparability of assessment methods if there is more than one group	10-11
Bias	9	Describe any efforts to address potential sources of bias	10
Study size	10	Explain how the study size was arrived at	8
Quantitative variables	11	Explain how quantitative variables were handled in the analyses. If applicable, describe which groupings were chosen and why	10
Statistical methods	12	(*a*) Describe all statistical methods, including those used to control for confounding	10-11
		(*b*) Describe any methods used to examine subgroups and interactions	11
		(*c*) Explain how missing data were addressed	10
		(*d*) If applicable, describe analytical methods taking account of sampling strategy	10
		(*e*) Describe any sensitivity analyses	10
**Results**			
Participants	13*	(a) Report numbers of individuals at each stage of study—eg numbers potentially eligible, examined for eligibility, confirmed eligible, included in the study, completing follow-up, and analysed	11
		(b) Give reasons for non-participation at each stage	11
		(c) Consider use of a flow diagram	23
Descriptive data	14*	(a) Give characteristics of study participants (eg demographic, clinical, social) and information on exposures and potential confounders	11
		(b) Indicate number of participants with missing data for each variable of interest	12
Outcome data	15*	Report numbers of outcome events or summary measures	12
Main results	16	(*a*) Give unadjusted estimates and, if applicable, confounder-adjusted estimates and their precision (eg, 95% confidence interval). Make clear which confounders were adjusted for and why they were included	12

For peer review only - http://bmjopen.bmj.com/site/about/guidelines.xhtml

		(*b*) Report category boundaries when continuous variables were categorized	12
		(*c*) If relevant, consider translating estimates of relative risk into absolute risk for a meaningful time period	NA
Other analyses	17	Report other analyses done—eg analyses of subgroups and interactions, and sensitivity analyses	13
**Discussion**			
Key results	18	Summarise key results with reference to study objectives	15
Limitations	19	Discuss limitations of the study, taking into account sources of potential bias or imprecision. Discuss both direction and magnitude of any potential bias	17
Interpretation	20	Give a cautious overall interpretation of results considering objectives, limitations, multiplicity of analyses, results from similar studies, and other relevant evidence	17-18
Generalisability	21	Discuss the generalisability (external validity) of the study results	17
**Other information**			
Funding	22	Give the source of funding and the role of the funders for the present study and, if applicable, for the original study on which the present article is based	19

*Give information separately for exposed and unexposed groups.

Note: An Explanation and Elaboration article discusses each checklist item and gives methodological background and published examples of transparent reporting. The STROBE checklist is best used in conjunction with this article (freely available on the Web sites of PLoS Medicine at http://www.plosmedicine.org/, Annals of Internal Medicine at http://www.annals.org/, and Epidemiology at http://www.epidem.com/). Information on the STROBE Initiative is available at www.strobe-statement.org.

For peer review only

For peer review only - http://bmjopen.bmj.com/site/about/guidelines.xhtml
